# Emerging Molecular Receptors for the Specific-Target Delivery of Ruthenium and Gold Complexes into Cancer Cells

**DOI:** 10.3390/molecules26113153

**Published:** 2021-05-25

**Authors:** João Franco Machado, João D. G. Correia, Tânia S. Morais

**Affiliations:** 1 Centro de Química Estrutural and Departamento de Química e Bioquímica, Faculdade de Ciências, Universidade de Lisboa, Campo Grande, 1749-016 Lisbon, Portugal; jmfmachado@fc.ul.pt; 2 Centro de Ciências e Tecnologias Nucleares and Departamento de Engenharia e Ciências Nucleares, Instituto Superior Técnico, Universidade de Lisboa, Estrada Nacional 10 (km 139, 7), 2695-066 Bobadela LRS, Portugal

**Keywords:** ruthenium, gold, targeted drug delivery, cancer, therapeutic targeting agents, precision medicine

## Abstract

Cisplatin and derivatives are highly effective in the treatment of a wide range of cancer types; however, these metallodrugs display low selectivity, leading to severe side effects. Additionally, their administration often results in the development of chemoresistance, which ultimately results in therapeutic failure. This scenario triggered the study of other transition metals with innovative pharmacological profiles as alternatives to platinum, ruthenium- (e.g., KP1339 and NAMI-A) and gold-based (e.g., Auranofin) complexes being among the most advanced in terms of clinical evaluation. Concerning the importance of improving the in vivo selectivity of metal complexes and the current relevance of ruthenium and gold metals, this review article aims to survey the main research efforts made in the past few years toward the design and biological evaluation of target-specific ruthenium and gold complexes. Herein, we give an overview of the inorganic and organometallic molecules conjugated to different biomolecules for targeting membrane proteins, namely cell adhesion molecules, G-protein coupled receptors, and growth factor receptors. Complexes that recognize the progesterone receptors or other targets involved in metabolic pathways such as glucose transporters are discussed as well. Finally, we describe some complexes aimed at recognizing cell organelles or compartments, mitochondria being the most explored. The few complexes addressing targeted gene therapy are also presented and discussed.

## 1. Introduction

Although metallodrugs play unique roles in the clinical setting, they are niche amongst the drug arsenal currently available for diagnostic or therapeutic applications. Apart from the metal-based radiopharmaceuticals used in nuclear medicine for diagnostic (e.g., ^99m^Tc or ^68^Ga) and/or therapeutic (e.g., ^90^Y or ^177^Lu) procedures and cisplatin (and derivatives) for cancer treatment, the number of approved metal-based drugs by the regulatory authorities is quite low. However, the potential of these compounds has not been fully explored yet in order to benefit from their particular chemical and physical properties. This may lead to the discovery of drugs with novel mechanisms of action, opening the possibility for addressing still unmet needs in the clinical arena. Considering that metal complexes are more versatile compared to pure organic molecules, mainly due to the various coordination states adopted by the metal centers and/or their important redox activity to mention a few specific features, metallodrugs can be fine-tuned to optimize biological interactions but also organ distribution and internalization by cancer cells [1,2].

Indeed, metal-based drugs, as therapeutic and/or diagnostic agents, may display innovative pharmacological profiles in relation to novel molecular mechanisms still poorly understood. However, there are still several relevant issues that need to be addressed in the quest for novel metal complexes with higher activity and selectivity, which would ultimately lead to effective metallodrugs with fewer undesirable side effects. Such concerns include the prejudice against metals due to toxicity issues observed in certain circumstances, which create resistances towards the approval of metal-based drugs by the medicine agencies; the low selectivity of metal complexes that leads to poor differentiation between healthy and diseased tissues and the low in vivo stability of the newly designed metal complexes, amongst others [3,4,5]. The latter question depends on the selection of the most adequate ligands as discussed in various review articles. Among the strategies explored in the last years to increase in vivo selectivity, the design of metal complexes bearing pendant moieties that recognize specifically and with high affinity targets that are related to a specific disease or disease state has received considerable attention. This approach has reached particular importance in the case of cancer, where several relevant biomarkers were identified, and some of them are important targets for in vivo molecular imaging and/or therapy [6,7,8,9,10]. For the sake of example, let us refer to antigens (e.g., CD20 or prostate-specific membrane antigen), membrane receptors (e.g., integrins, G-protein coupled receptors, or epidermal growth factor receptor), and enzymes (e.g., carbonic anhydrases or thymidine kinases). These biomarkers play important roles in pathophysiological processes, in most cases being overexpressed or upregulated in cancer cells compared to the expression levels of endogenous normal cells. Moreover, the fast-growing tumor cells depend on high levels of energy and nutrients, such as glucose, amino acids, or vitamins. Consequently, they show an altered metabolism compared to normal cells. The higher level of tumor vascularization through neoangiogenesis and the overexpression of transporters at cancer cell surfaces (e.g., glucose transport protein Glut1) compared to the healthy tissues contribute to a higher rate of nutrients apport by the tumor tissues. Therefore, transporter-targeted anticancer therapeutic approaches have been developed based on these metabolic differences [11,12,13,14]. 

As regards the pendant target-specific moieties mentioned above, monoclonal antibodies or antibody fragments could be considered quite relevant options considering their exquisite specificity towards the corresponding disease-specific antigen [15,16]. However, as far as we are aware, there are not many reported examples of their use as vectors of metal complexes. The most studied and explored entities for the selective delivery of metal complexes are, undoubtedly, small molecules and, in the majority of cases, peptides. Indeed, following the finding that small endogenous regulatory peptide receptors are often overexpressed in human cancers and that derivatives of their natural ligands can be used for tumor targeting, the use of peptides has emerged as an important approach for selective delivery. This strategy has mainly been driven by the successful accomplishments in diagnostic imaging and peptide receptor radionuclide therapy (PRRT) within the framework of nuclear medicine [17,18,19]. 

**Figure 1:**
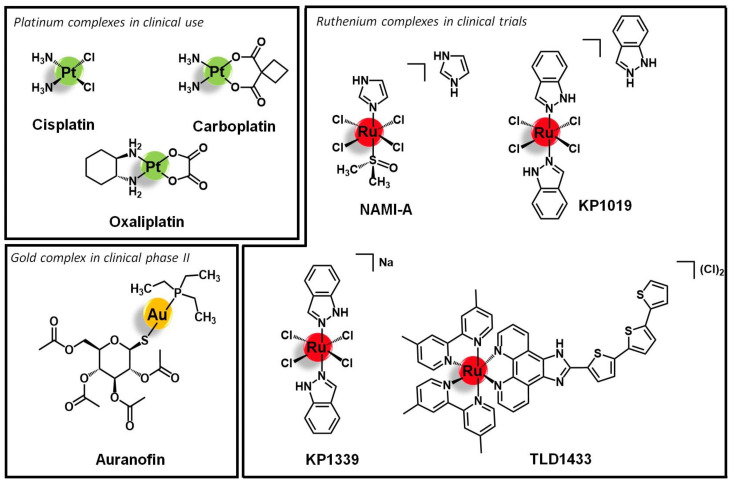
Selected platinum, ruthenium, and gold anticancer lead structures.

The metal cores directly responsible for the antiproliferative/cytotoxic activity, cisplatin, and derivatives (Figure 1) are still the paradigmatic examples of the application of metal complexes in cancer therapy. In fact, all current research efforts have been developed towards replicating and improving the success of this approved family of metallodrugs. In particular, the main goal is bringing metal complexes into the next level in the therapeutic arsenal, where, beyond efficacy, selectivity and specificity are mandatory, especially when the new paradigm in medicine, precision medicine, is taken into consideration. Although highly effective in the treatment of certain cancers, cisplatin and derivatives do not fulfil the desired requisites, particularly in terms of selectivity. Indeed, these molecules display low selectivity leading to severe side effects. Additionally, several cancer cell lines show resistance against those molecules. This scenario prompted the study of other transition metals as alternatives to platinum-based metallodrugs, namely titanium, iron, osmium, rhodium, iridium, palladium, platinum, ruthenium, and gold [20,21,22,23,24,25].

Ruthenium and gold complexes are among the most investigated and advanced non-platinum based metallodrugs in terms of clinical evaluation and the study of the respective mechanisms of action. To date, four Ru complexes, namely KP1019 and its sodium analog KP1339 as well as NAMI-A and TLD1433 (Figure 1), have entered clinical evaluation as systemic anticancer therapeutics [2,26,27,28,29,30,31,32,33]. Gold complexes emerged as well as suitable antiproliferative agents due to their mechanism of action [3,4,5,34]. Indeed, Au(I) complexes selectively target enzymes bearing residual thiol or selenol groups and Au(III) is isoelectronic to Pt(II) known from cisplatin. Additionally, in multi-metallic complexes, the nuclei can form aurophilic interactions leading to higher stability and ideally to luminescence properties. Auranofin (Figure 1) is the leading gold compound that has been studied in clinical phase II against chronic lymphocytic leukemia [35].

**Figure 2:**
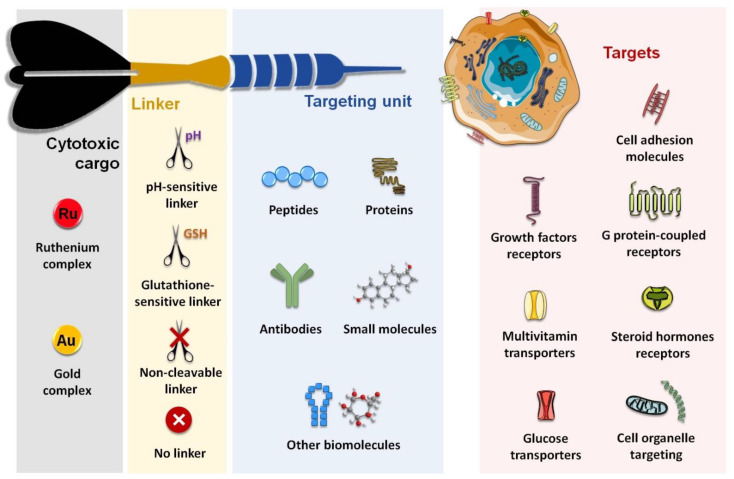
Schematic structure of receptor targeting Ru and Au conjugates.

Considering both the importance of improving the in vivo selectivity of metal-based complexes discussed above and the relevance reached by ruthenium- and gold-based complexes within the context of innovative anticancer agents, this article aims to review the main research efforts made in the past few years towards the design and biological evaluation of target specific ruthenium and gold complexes. The main general characteristics of these complexes are depicted in Figure 2.

We will give an overview of the extensive number of inorganic and organometallic molecules that have been conjugated to different biomolecules for targeting membrane proteins, namely cell adhesion molecules (integrins and cadherins), G-protein coupled receptors (somatostatin receptors, bombesin receptors, and opioid receptors), and growth factor receptors (epidermal growth factor receptor, human epidermal growth factor receptor 2, and fibroblast growth factor receptor). We will also refer to complexes that recognize emerging targets such as the case of progesterone receptors or those involved in metabolic pathways such as glucose transporters (e.g., Glut1). Finally, we will describe some complexes that were aimed at recognizing cell organelles or compartments, mitochondria, considered the “powerhouse” of the cell, being the most explored. The few complexes addressing targeted gene therapy are also briefly described.

## 2. Cell Adhesion Molecules (CAM)

Cell adhesion molecules (CAM) are cell surface glycoproteins involved in cell-to-cell and cell-to-extracellular matrix adhesion, a process that is essential for the correct maintenance and function of tissues and organs [36]. CAM are grouped into four different classes—integrins, cadherins, selectins, and the immunoglobulins superfamily. While integrins typically bind to the extracellular matrix, the other three types of CAM are usually associated with cell-to-cell adhesion phenomena [36]. Additionally to their structural function, CAM also act as receptors of a variety of endogenous ligands and messengers, modulating and actively participating in different key biological processes, including cell proliferation and migration, phagocytosis, apoptosis, angiogenesis, and thrombosis [36,37]. Alterations of CAM function, structure, and/or expression patterns are often associated with auto-immune diseases, metabolic syndromes, and cancer [36]. Therefore, CAM have been intensively exploited as potential drug targets, and for instance, some CAM-targeting drugs were already approved for the treatment of patients with thrombosis. Currently, there are also several drug candidates under clinical trials for the treatment of cancer and other disorders [36,37]. Moreover, CAM have also been exploited as targets for targeted drug delivery in precision medicine, given the overexpression of specific CAM in certain diseases, such as cancer, comparatively to healthy tissues. This approach relies on the use of ligands/targeting units (e.g., antibodies, peptides, peptidomimetics, and small molecules, among others) that can recognize and bind with high affinity to the specific type of CAM that is overexpressed in the surface of the tumoral cells as drug carriers, thus being able to selectively delivery the drug into its target while sparing the surrounding tissues [37]. Within this frame, substantial work has been continuously reported, especially regarding the use of integrin- and cadherin-targeting peptides [36].

### 2.1. Integrins

**Figure 3:**
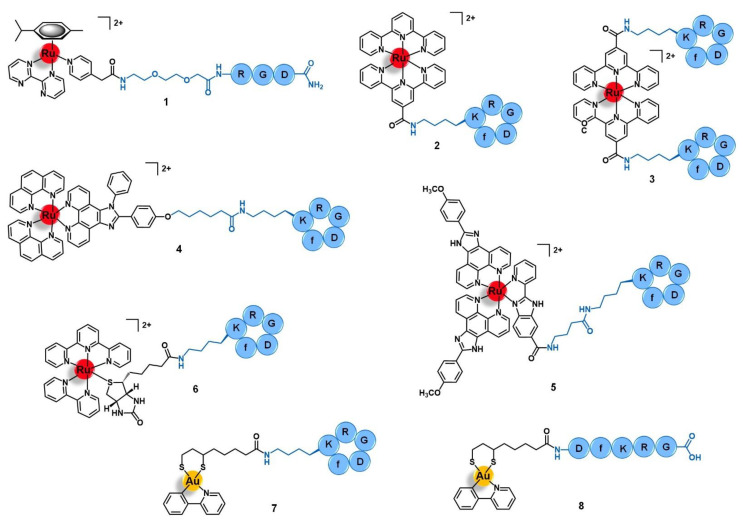
Ru(II)- and Au(III)-conjugates containing an integrin-targeting peptide as the delivering agent of the active metal complex into cancer cells: conjugates with the peptides RGD (**1**), *cyclo*-RGDfK (**2** to **7**), and DfKRG (**8**). Note: f = D-phenylalanine.

Integrins are heterodimeric transmembrane receptors composed of an α- and a β-subunit non-covalently associated with each other, that are dependent of divalent cations such as Mg^2+^ or Ca^2+^ for the interaction with their ligands [38,39]. There are 24 subtypes of integrins in mammals, resulting from a limited number of combinations between 18 different α-subunits and 8 diverse β-domains [38]. Additionally to these structural differences, each subtype also has its own cellular distribution, endogenous ligands (e.g., collagen, fibronectin, nephronectin, laminin, etc.), and function [39]. In general, integrins play an important role in a plethora of biological processes by acting as adhesion molecules, mechanosensors, and signal transduction platforms. They can signal both from the extracellular environment into the intracellular compartment as well as in the opposite sense, regulating cell adhesion, proliferation, migration, and survival [38,39]. Integrins also mediate several cancer-related events, such as tumor initiation and progression, malignant transformation, tumor-induced angiogenesis, cancer metastasis and reactivation, and resistance to anticancer immunotherapy [40]. Given the correlation of integrins with the etiology and pathology of several diseases, various integrin-targeting drugs have successfully achieved clinical use and many others are under clinical development, most of them aiming to treat cardiovascular diseases, auto-immune syndromes, and cancer [41]. In the latter, integrins such as α_V_β_3_ and α_V_β_5_ are upregulated in certain types of tumors relative to the other non-tumoral cells, displaying a characteristic distribution in cancer tissues, and/or structural alterations during tumor growth and metastasis [40,42]. Thus, integrins have also been exploited for the targeted drug delivery of anticancer agents by using drug carriers that can selectively bind to these receptors and trigger an integrin-mediated endocytosis process with subsequent accumulation of the drug specifically in the tumor cells [43]. The arginine-glycine-aspartic acid (RGD) motif was found to be present in several natural ligands of the α_V_-integrin subfamily (such as fibronectin) and to be the minimal sequence needed for appropriate integrin recognition [43,44]. Thus, peptides containing the RGD motif have become a popular tool to the selective delivery of known drugs or drug candidates, including organic small molecules and metal complexes, into integrin-expressing cancer cells for precision therapy and diagnostics [18,19]. Several linear and cyclic peptides have been custom designed as highly specific binders of α_V_β_3_, α_V_β_6_, or α_5_β_1_ integrins and many studies regarding their use as delivering vectors for anticancer applications have been reported with promising results [43,44]. However, only few studies of ruthenium and gold complexes vectorized with integrin-targeting agents have been reported. Most of them address the use of the cyclic peptide *cyclo*-RGDfK (f = D-phenylalanine) that is known to bind selectively and with high affinity to the α_V_β_3_ integrin, but the linear tripeptide RGD (specific for α_V_β_3_/α_V_β_5_) and other RGD-containing sequences were exploited as well. Marchán and co-workers reported the conjugation of the complex [Ru(η^6^-*p*-cym)(bpm)(pyac)]^2+^ (where *p*-Cym = *para*-cymene; bpm = 2,2′-bipyrimidine; and pyac = 4-pyridineacetic acid) with the tripeptide RGD using a polyethylene glycol spacer between both moieties (**1**, Figure 3) [45]. The spacer PEG(2) was selected to improve the aqueous solubility of the conjugate and to keep the ruthenium complex spatially apart from the targeting-peptide so that the activity and the selectivity of each would not be perturbed. Conjugate **1** acts as a prodrug that is stable in aqueous solution at dark, but upon visible light irradiation suffers selective photodissociation from the pyridyl-RGD functionalized ligand, releasing the active complex [Ru(η^6^-*p*-cym)(bpm)(H_2_O)]^2+^ [45]. Other authors have explored the conjugation of peptide *cyclo*-RGDfK with different ruthenium-polypyridyl complexes for applications in targeted therapy and/or diagnostics of human breast adenocarcinoma, glioblastoma, cervical cancer, and head and neck tumors [46,47,48]. Kühn and co-workers studied the vectorization of a terpyridine-based ruthenium complex towards α_ν_β_3_-expressing cancer cells by using one or two *cyclo*-RGDfK peptides [46]. Conjugates **2** and **3** (Figure 3) were synthesized via amide bond formation between the amine group present at the sidechain of the lysine residue of the targeting-peptide and the carboxylic acid group of the precursor complexes [Ru(terpy)(terpyCOOH)]^2+^ or [Ru(terpyCOOH)_2_]^2+^, respectively (where terpy = 2,2′:6′,2′′-terpyridine; terpyCOOH = [2,2′:6′,2′′-terpyridine]-4′-carboxylic acid). Both conjugates showed high affinity and selectivity towards the α_ν_β_3_ integrin (IC_50_ **2** = 49 nM; IC_50_ **3** = 2.5 nM) as compared to its α_V_β_5_ analogue (IC_50_ **2** > 1000 nM; IC_50_ **3** = 595 nM). The 20-fold higher affinity displayed by the dipeptide conjugate **3** comparatively to the mono-derivatized one emphasizes the role of *cyclo*-RGDfK as the targeted delivery agent. However, both conjugates showed low in vitro cytotoxicity (IC_50_ values > 85 μM) against both cell lines with scarce expression of α_ν_β_3_ (A549, human non-small-cell lung cancer) or moderate expression of this receptor (SKOV3, human mammary carcinoma), with no significant difference between them. The poor antiproliferative activity of **2** and **3** is attributed to the intrinsic lack of cytotoxicity of the free ruthenium complexes and to their low uptake by the cancer cells despite the increased affinity of the conjugates towards the integrin receptors [46].

The photodynamic therapy (PDT) of cancer is a non-invasive approach based on the use of photoactivable sensitizers to elicit a local anti-tumor response upon specific light irradiation [47]. 

Aimed at the development of a new precise photodynamic therapeutic approach against human glioblastoma, Wang et al. vectorized the complex [Ru(phen)_2_(phenimi)]^2+^ (phen = 1,10-phenanthroline; and phenimi = 6-(4-(1-phenyl-1*H*-imidazo[4,5-*f*][1,10]phenanthrolin-2-yl) phenoxy)hexanoic acid) with *cyclo*-RGDfK (**4**, Figure 3) [48]. Conjugate **4** selectively targets the mitochondria of α_V_β_3_-overexpressing glioblastoma cells and induces cell death under appropriate light irradiation conditions, both in vitro and in vivo. It preferentially accumulates in α_V_β_3_-positive cancer cells (U87MG, human glioblastoma) rather than in α_V_β_3_-negative cancer cells (MCF-7, human breast adenocarcinoma) with a higher degree of cellular uptake than the respective non-vectorized ruthenium complex in U87MG cells, but a similar degree of internalization in MCF-7 cells. The selective uptake of **4** in U87MG cell lines seems to be mediated by the α_V_β_3_ integrins by blocking assays with the RGD tripeptide in this cell line. After internalization by U87MG cells, conjugate **4** selectively accumulates in the mitochondria (85%) and cytosol, being excluded from the nucleus. Moreover, contrary to the non-vectorized complex, the conjugate was shown to have a selective cytotoxic action in vitro, being more active upon irradiation than in dark conditions against the α_V_β_3_(+) cells and without significant cytotoxicity against the αVβ3(−) cell line, either in the dark or upon irradiation. Further in vitro studies with 3D multicellular tumor spheroids of U87MG cells showed that **4** has deeper tissue penetration and was able to reduce the diameter of the spheroids over time, showing suitable characteristics for the PDT of deep tissues. Regarding the mechanism of action, in this 3D model and upon light irradiation, the conjugate induced the production of reactive oxygen species (ROS) and induced cell death by apoptosis mediated by mitochondria-dependent signaling pathways. In vivo, it showed a remarkable inhibition of tumor growth upon two-photon PDT of U87MG tumor-bearing Balb/c mice with a tumor inhibition rate of 87% compared to the non-vectorized complex (15%) in the same conditions. Without the two-photon PDT, the conjugate only showed a rate of 29%. Moreover, conjugate **4** preferentially accumulated in the tumor rather that in the main organs of the mice, in contrast with the non-vectorized complex that was found in a higher content at the liver than in the tumor. The four-fold higher accumulation of **4** in the tumor compared to the respective non-conjugated complex is consistent with the higher cellular uptake observed and might explain the high anticancer activity together with no significant damage of the remaining healthy organs. Altogether, these results suggest that conjugate **4** has favorable properties to targeted photodynamic therapy and, according to the authors, holds the potential to be further developed as a multifunctional mitochondria-targeting agent in cancer theranostics [48].

It is known that cancer tissues have a typical microenvironment around them that differs from the healthy state, including different pH, oxygen levels, redox potential, intra and extracellular enzymes, etc. Many authors have been exploring the use of drug-delivery systems responsive to cancer-dependent stimulus for a precise and controlled release of the drug into the tumor, while remaining inert during body distribution and after reaching non-tumoral organs [49]. Chen’s group reported a ruthenium-*cyclo*-RGDfK prodrug (**5**, Figure 3) that is pH-sensitive to the acidic tumor’s microenvironment (≈ 6.5 to 6.9) and that could be potentially useful as a theranostic agent against cervical cancer [50]. Conjugate **5** was prepared by a condensation reaction between the lysin residue of *cyclo*-RGDfK and the carboxylic acid group of the luminescent complex [Ru(POP)_2_(pbiz)]^2+^ (POP = 2-(4-methoxyphenyl)imidazo[4,5-f]1,10-phenanthroline; pbiz = 2-(pyridin-2-yl)-1*H*-benzo[d]imidazole-6-carboxylic acid). Conjugate **5** showed higher in vitro uptake by CaSki, SiHa, and HeLa cervical cancer cell lines via α_ν_β_3_ integrin receptor-mediated mechanism and higher cytotoxicity than the respective non-conjugated ruthenium complex, inducing cell death by apoptosis. Furthermore, it was also shown to be less cytotoxic in other cell lines with lower expression of α_ν_β_3_ integrins (e.g., MCF-7 human breast cancer cells, Ect1/E6E7 non-tumoral cervical cells, and L02 human hepatocytes) with a safety index up to five-fold higher. Conjugate **5** is stable in solutions at physiological pH (7.4) over 24 h, however, at pH < 6.8 (tumor microenvironment), it suffers hydrolysis, with substitution of the pbiz ligand by two water molecules, releasing complex [Ru(POP)_2_(H_2_O)_2_]^2+^ from the targeting peptide. This activated aqueous Ru complex exhibits a cytotoxicity of the same order of magnitude as the non-vectorized Ru complex against the same cancer cell lines, and therefore might correspond to the active drug obtained after the conjugate reaches the tumor. Additionally to these promising results, conjugate **5** also demonstrated favorable deep-red luminescent properties after one-photon and two-photon excitation, allowing the deep tissue imaging of 3D tumor spheroids of CaSki cells. The group of Chen et al. also studied the biodistribution and the potential therapeutic effect of **5** in CaSki-inoculated xenograft mice. Interestingly, 36 h after administration of **5** (4 μmol/kg) there was a selective tumor accumulation which allowed imaging it. In contrast, the non-conjugated Ru complex was distributed non-specifically through the animal, accumulating in the liver, spleen, lung, and kidney. Remarkably, after 25 days of treatment (12 doses at 4 μmol/kg), there was a considerable tumor weight reduction (74%) without the appearance of pathological damage or abnormalities of the healthy tissues. Unlike the promising results obtained with **5**, the non-conjugated complex only gave a tumor reduction of 53% and led to spleen damage. In addition, after 25 days of treatment, the tumor induced liver and renal dysfunctions in the mice treated with the free complex, but those treated with the conjugate had their kidney and liver functions back to normal after the same period. Ex vivo imaging studies of cervix tumoral and non-tumoral tissue samples from 38 human patients were also performed. Unlike the non-conjugated complex, conjugate **5** was able to distinguish healthy tissues from the tumoral ones, as well as identify cervical cancer at different stages with a sensitivity of 95% and a specificity of 100%. Owing to the promising results, the authors pointed to this conjugate as a pH-responsive delivery system able to release a luminescent and cytotoxic ruthenium complex in a controlled way after activation by the acidic microenvironment of the tumor, rendering it a potential multifunctional theranostic agent for application in the precise therapy of cervical cancer [50].

The use of ruthenium conjugates for ex vivo targeted cancer diagnosis was exploited by Casini and co-workers who reported the synthesis of conjugate **6**, which was obtained by conjugation of the photocleavable complex [Ru(terpy)(bipy)(D-biotin)]^2+^ (bipy = 2,2′-bipyridine) to *cyclo*-RGDfK (Figure 3). Conjugate **6** was used as a mass-tag for potential application in targeted epitope-based laser desorption ionization mass spectrometry imaging (LDI-MSI) of hypopharyngeal squamous cell carcinoma [51]. LDI-MSI is a technique based on using laser cleavable mass-tags that bind specifically and with high affinity to given moieties present in the tissues under analysis for the detection of proteins of interest, such as the α_V_β_3_ integrins in this case. The latter shows a characteristic distribution pattern in hypopharyngeal carcinomas, compared to healthy organs, allowing the diagnostics. Conjugate **6** binds to α_V_β_3_ integrins with high affinity (IC_50_ = 3.2 nM) and selectivity (IC_50_ for α_5_β_1_ and α_V_β_5_ ≈ 500 nM), a characteristic that together with its photocleavable properties render the conjugate suitable as a probe for matrix-free LDI-MSI. Inside the mass spectrometer ionization chamber, conjugate **6** can be cleaved from its molecular target on the cancer tissue surface sample upon UV-light irradiation, which releases a fragment identified as [Ru(terpy)(bipy)(pyridine)-3*H*]^+^. The latter provides a fingerprint signal in the MS spectrum with specific mass and isotopic pattern distribution that allows its unambiguous identification for indirect target detection. Moreover, incubation of the cancer tissue section with **6** allowed to clearly distinguish the signal corresponding to the distribution of the mass-tag with a pattern that correlated with the distribution of the α_V_β_3_ integrins determined by classical methods of immunohistochemistry and hematoxylin staining. On the other hand, incubation with the non-conjugated complex resulted in unspecific and scarce detection by LDI-MSI. Given these results, the authors suggested that conjugate **6** holds potential to be employed as a sensitive tool for matrix-free targeted LDSI-MSI and that further modifications of the Ru fragment and/or of the targeting moiety would eventually allow far-reaching applications in cancer diagnostics [51].

Concerning gold complexes, only a few examples of target-delivery using integrin-binding peptides have been reported. Recently, Metzler-Nolte and colleagues developed two Au(III)-peptide conjugates based on the complex [Au(ppy)(Lpa)] (ppy = 2-phenyl-pyridine; and Lpa = lipoic acid) with the *cyclo*-RGDfK (**7**, Figure 3) and the linear DfKRG peptides (**8**, Figure 3) [52]. The natural product Lpa was chosen as a tethering moiety given its chemical structure and for being known for its own anticancer properties. Both conjugates were prepared by firstly derivatizing the peptides with Lpa, followed by reducing Lpa’s internal disulphide bond, and then reacting it with the complex [Au(ppy)Cl_2_]. Conjugate **7**, containing *cyclo*-RGDfK, showed an eight-fold increased cytotoxicity in vitro against MCF-7 and MDA-MB-231 human breast cancer cell lines compared to the parent complex [Au(ppy)Cl_2_]. The conjugate with the linear DfKRG peptide (**8**) was shown to be less cytotoxic than the cyclic analogue (ca. 18-fold less active) and even less cytotoxic than the non-vectorized gold complex (ca. 3-fold less active). These results suggest that the use of integrin-targeting peptides as carriers might be a promising approach to the targeted delivery of gold complexes into breast cancer cells [52].

Overall, despite the use of integrin-targeting vectors for the precise delivery of cytotoxic complexes of ruthenium and gold into cancer cells being still in the very beginning of preclinical evaluation, the preliminary results suggest that this might be a successful strategy for the treatment and/or diagnosis of several types of tumors, either applied in single or in combined therapy with other well-stablished approaches such as PDT.

### 2.2. Cadherins

Although less exploited than integrins, cadherins are another class of CAM that has been explored for specific delivery of anticancer agents into tumors [37]. Cadherins are calcium-dependent adhesion glycoproteins associated with the cell-to-cell adherent junctions on solid tissues, in which they mediate and regulate the reorganization of the cell cytoskeleton, intracellular signaling, and transcriptional regulation processes, as well as angiogenesis, morphogenesis and tissue growth, differentiation, and organization [53]. Cadherins are grouped into three main families—classical cadherins (type I and II), protocadherins, and atypical cadherins. The largest and most studied family, classical cadherins are highly conservative structures expressed in a tissue-specific manner and are subclassified according to the location they are typically associated with, for example, neural (N)-, epithelial (E)-, placental (P)- and vascular endothelial (VE)-cadherins [53]. Alterations in cadherin-mediated processes are often associated with cancer growth and dissemination. For instance, many tumors go through a phenomenon called cadherin switch, in which N-cadherins are upregulated while E-cadherins are downregulated, inducing tumor cells to resist natural apoptosis and gain invasive and metastatic capacity [54,55]. Consequently, E- and N-cadherins have both been exploited as targets for cancer therapy, with some drug candidates achieving clinical trials [54,55]. The overexpression of cadherins in several types of cancer cells compared to the remaining non-tumoral tissues and their role in the permeation of biological barriers through the paracellular pathway have also opened the possibility of exploring them as receptors for targeted drug delivery [54,55,56]. A common approach involves the use of antagonist peptides containing the histidine-alanine-valine (HAV) sequence, as this motif corresponds to the cell adhesion recognition sequence present in the extracellular subdomain EC1 of cadherins that is essential for their correct adhesion and function. HAV-based peptides can bind selectively and with high affinity to cadherins, thus being used either as targeted anticancer agents or targeted drug delivery carriers [55,56].

**Figure 4:**
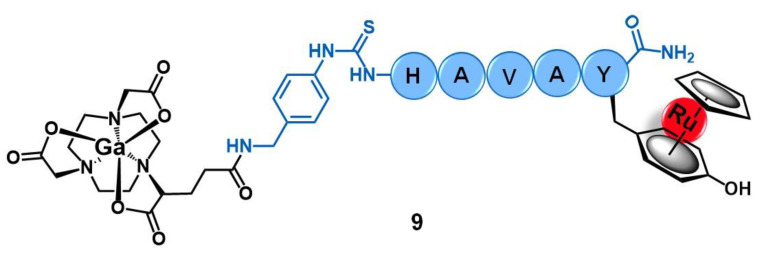
Ru(II)/Ga(III) heterobimetallic conjugate with the cadherin-targeting peptide HAVAY (**9**).

Buglyó and co-workers reported the first heterobimetallic conjugate with an HAV-based peptide for application in cancer theranostics [57]. The peptide-containing radioactive complex ^67^Ga-NODAGA-[(η^6^-Tyr-RuCp)-HAVAY-NH_2_] (**9**, Figure 4, Cp = η^5^-C_5_H_5_; HAVAY = his-ala-val-ala-tyr; and NODAGA = 2,2′-(7-(1-carboxy-4-((4-isothio cyanatobenzyl)amino)-4-oxobutyl)-1,4,7-triazonane-1,4-diyl)diacetic acid) was prepared by metalation of the HAVAY at its tyrosine residue with [RuCp(η^6^-naphthalene)] under visible-light irradiation, forming a sandwich-type Ru(II) complex, followed by conjugation to NODAGA and labeling with ^67^Ga(III). Conjugate **9** was designed aiming to contain three functional moieties for targeted theranostics: i) the HAVAY peptide to target the cadherins overexpressed at cancer cells and thus acting as a drug carrier, ii) a Ru(II) complex with potential anticancer activity, and iii) a ^67^Ga-radiolabeled core (γ-emitter) for imaging purposes by single photon emission computed tomography (SPECT). The cellular uptake of the conjugate was determined in four human cancer cell lines with different expression levels of N-/E-cadherins, namely A375(+/−) melanoma, PC-3(+/+) prostate, and MCF-7(−/+) and MDA-MB-231(−/−) breast cancer cells. Surprisingly, conjugate **9** showed low to moderate uptake, not related to the cadherins expression levels, with the highest uptake rate found in MDA-MB-231 cells (14.9%) in which the conjugate was mostly retained at the cell membrane. Additionally, in this cell line, the conjugate also was not shown to be cytotoxic, most probably due to the low cellular internalization and retention [57].

Even though cadherins are still sparsely exploited as targets for the specific delivery of ruthenium and gold complexes into cancer cells, with plenty of room for further studies aiming to optimize and take full advantage of this approach, we believe that it might become a promising strategy given the good results found for other classes of cell-adhesion molecules (such as integrins, see previous section).

## 3. G Protein-Coupled Receptors (GPCR)

G protein-coupled receptors (GPCR) are the largest family of cell transmembrane proteins encoded by humans, and are responsible for transducing a variety of extracellular stimuli into a plethora of key physiological processes by initiating complex and diverse intracellular signaling cascades [58,59]. With over 800 different GPCR, they are divided into four main groups according to their pharmacological properties: rhodopsin-like (class A), secretin-like (class B), metabotropic glutamate-like (class C), and frizzled receptors (class D). Despite their different functions and amino-acid sequence diversity, all GPCR display a characteristic common structural feature, namely a transmembrane domain essential for signal transduction composed of 7 helices embedded in the cell membrane, connected through 3 extracellular and 3 intracellular loops [58]. The GPCR’s extracellular endogenous ligands are very diverse as well, ranging from small molecules to large proteins, including chemokines, neurotransmitters, hormones, and lipids, among many others. Upon binding, these ligands induce GPCR conformational changes, allowing signal transduction by interaction with G protein and other intracellular binders [58,59]. These receptors have been associated with a large number of diseases, including obesity, metabolic syndromes, neuronal disorders, and cancer. Therefore, they have been intensively exploited as drug targets [59,60]. Indeed, approximately one third of clinically approved drugs target these receptors and a continuously increasing number of GPCR-targeting drug candidates are under clinical trials and under pre-clinical development [60]. Additionally, a rising trend of exploring GPCR in precision medicine for targeted therapy, diagnostics, and/or drug delivery has been reported as well [60,61]. Within this context, in this section, we discuss the most relevant GPCR that have been studied for the targeted-delivery of ruthenium and gold complexes into cancer cells, including the somatostatin receptors (SSTR), bombesin receptors (BBR), opioid receptors (OPR), and G protein-coupled estrogen receptors (GPER), all of them belonging to the Class A group.

### 3.1. Somatostatin Receptors (SSTR)

Somatostatin receptors comprise five subtypes in humans (SSTR1 to SSTR5), with different distributions throughout the central and the peripheral nervous systems [62]. They act as receptors of endogenous neuropeptides, such as the cognate hormone somatostatin (also known as somatotropin release inhibiting factor) and cortistatin. SST receptors are responsible for modulating neuronal activity and the levels of several neuronal and growth hormones, including the somatotropin [62,63]. It is known that many types of cancers (e.g., lung, breast, prostate, adrenal, and neuroendocrine cancers) and tumor blood vessels overexpress these receptors, especially SSTR2, compared to the non-tumoral tissues [62,63]. Thus, many anticancer and antiangiogenic drugs have been vectorized into tumors by SSTR-targeting peptides [63]. The endogenous somatostatin, a 16-residues length cyclic peptide of sequence AGCKNFFWKTFTSC with a disulphide-bond between the two cysteines, was one the first vectors studied. However, many somatostatin analogues have also been reported, and special relevance has been attained by the cyclic peptide octreotide (8-residues length sequence fCFwKTCT, f = D-phenylalanine and w = D-tryptophane, with a disulphide bridge between its cysteines), as it shows higher stability under physiological conditions than somatostatin, allied to a higher selectivity towards SSTR2 subtype [63]. For both somatostatin and octreotide, the common approach for conjugation of the peptides to the delivering cargo via a lysin residue cannot be used, as this residue is part of the SSTR binding domain, and its modification hampers the receptor’s recognition [62,63].

**Figure 5:**
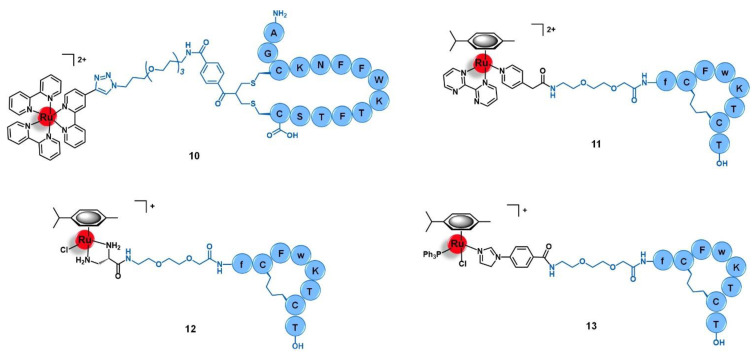
Ru(II) conjugates that target the somatostatin receptors (SSTR) have as vectors peptides derived from somatostatin (**10**) or octreotide (**11** to **13**). Note: f = D-phenylalanine; w = D-tryptophane.

Aiming to develop a targeted anticancer photodynamic therapy (PDT) agent, Weil and co-workers tethered the somatostatin peptide to an alkyl-derivative of the photoactivable complex [Ru(bipy)_3_]^2+^ by copper catalyzed alkyne-azide cycloaddition (CuAAC) click reaction through an appropriate bis-alkylating linker containing an azide group, giving conjugate **10** (Figure 5) [64]. The selected linker allows the conjugation to somatostatin by disulphide rebridge, providing a facile approach to access defined conjugates that retain the structural conformation of the peptide and its capacity to bind the receptor. Additionally, the disulphide bond also renders the conjugate responsive to the high levels of glutathione (GSH) characteristic of cancer cells, which dissociates the conjugate and releases the active complex, consisting thus in a valuable strategy of targeted drug delivery dependent on tumor stimulation. The photostable conjugate **10** showed high activation of the somatostatin receptor (EC_50_ = 319.6 nM) in CHO-K1/Ga15/SSTR2 Chinese hamster ovary cells overexpressing the SSTR2, without signs of activation in the relative non-overexpressing wild-type cells CHO-K1/Ga15. Furthermore, in A549 human non-small-cell lung cancer cells that naturally overexpress SSTRs, the conjugate was rapidly transported across the membrane by endocytosis with an efficient accumulation inside the cells that were revealed to be 100-fold higher than the respective non-conjugated ruthenium complex. Moreover, in this cell line, **10** showed high cytotoxicity upon light irradiation via generation of ^1^O_2_ (IC_50_ = 13.2 mM) but remained nontoxic in the absence of irradiation (IC_50_ = 300 mM, phototoxic index = 23-fold). The non-conjugated Ru complex was shown to be five-fold less active than the Ru-peptide conjugate (IC_50_ = 67.5 mM), underling the importance of the increased cellular uptake given by somatostatin. Considering the good results against lung cancer cell lines, further work was performed aiming to explore the therapeutic potential of conjugate **10** in the treatment of acute myeloid leukemia (AML) [65]. In HL60 human leukemic cells, conjugate **10** also showed a higher uptake and cytotoxicity upon light irradiation (IC_50_ = 47.4 μM) than the respective peptide-free complex (IC_50_ > 100 μM), with a phototoxic index higher than 2. This conjugate preferentially accumulates in the lysosomes over other cellular organelles and generates reactive oxygen species (7.4-fold increase) that mediate cell death by apoptosis. In three AML cell lines (OCI-AML3, HL60, and THP-1), conjugate **10** showed 92 to 99% decrease in clonogenic growth comparatively to CD34+ enriched cord blood (CD34+ CB) cells used as control. Additionally, in primary AML cells collected from human patients with different levels of SSTR2 expression, conjugate **10** showed 74 to 99% reduction of their clonogenic capacity in 5 of 6 patient samples (with a minor response of 45% reduction in the 6th sample), demonstrating its in vitro potential to eradicate leukemic stem cells, which are responsible for the appearance and propagation of AML disease [65].

Marchán et al. conjugated a dicarba analogue of the cyclic peptide octreotide fCFwKTCT, in which the S-S bond was substituted by a CH_2_-CH_2_ bridge, to three ruthenium organometallic complexes aiming to target the SSTR2 receptors overexpressed at the membrane of tumoral cells [45,66]. Similarly to the analogous **1** (Section 2.1), [Ru(η^6^-*p*-cym)(bpm)(pyac)]^2+^ was conjugated to the dicarba octreotide through the PEG(2) spacer (**11**, Figure 5) [45]. Identical results were found as well, as **11** showed identical photoactivable properties and aqueous behavior, being able to interact specifically with DNA over other biological targets after its release from the targeting peptide upon light irradiation. The conjugation of the same dicarba-octreotide peptide to complexes [Ru(η^6^-*p*-cym)(dap)Cl]^+^ (dap = 1-(carboxylic acid)-1,2-diaminoethane; conjugate **12**, Figure 5) and [Ru(η^6^-*p*-cym)(PPh_3_)(imbez)Cl]^+^ (imbez = 4-(1H-Imidazol-1-yl)benzoic acid; conjugate **13**, Figure 5) also did not perturb the peptide structure arrangement nor the ability to recognize the SSTR, resulting in an increase of the cellular uptake in MCF-7 breast cancer and DU-145 prostate cancer cell lines that seems to be actively mediated through this receptor [66]. Conjugate **12** is hydrolyzed in an aqueous solution and forms adducts with DNA. In contrast, **13** was revealed to be inert to ligand substitution in solution, having no ability to bind this molecular target. However, conjugation of the complexes to the peptide moiety leads to reduced or even to complete loss of cytotoxicity. In MCF-7 breast cancer and DU-145 prostate cancer cell lines, **12** was not active, while **13** showed moderate-low cytotoxicity (IC_50_ MCF-7 = 63.0 μM; IC_50_ DU-145 = 26.0 μM). The lack of activity of complex [Ru(η^6^-*p*-cym)(dap)Cl]^+^ and its conjugate (**12**) could be eventually explained by the high hydrolysis rate of the Ru−Cl bond, leading to non-cytotoxic species. Despite the increase in cellular uptake, conjugate **13** showed 19-fold less activity in the breast cancer cells and 4-fold less in the prostate cancer line compared to the respective non-conjugated complex. Additionally, conjugate **13** did not show significant capacity to distinguish between cancer and non-cancer cell lines (IC_50_ non-tumoral CHO ovarian cells = 45.2 μM). Altogether, the results suggest that despite being possible to increase the cellular uptake of complexes with a SSTR-targeting peptide carrier, this is not enough per se to increase their cytotoxicity towards cancer cells that overexpress these receptors, but the intrinsic anticancer activity of the complexes and their ability to be released from the carrier and thus interact freely with the respective molecular target are of need as well [66].

### 3.2. Bombesin Receptors (BBR)

Bombesin receptors (BBR) are divided into three different subtypes, according to their endogenous ligands, namely the neuromedin B receptor (BBR1), the gastrin-releasing peptide (GRP) receptor (BBR2), and the orphan receptor class 3 for which the natural ligand is still unknown (BBR3) [67]. In humans, the endogenous analogue of the bombesin peptide (EQRLGNQWAVGHLM) is the GRP hormone, a 27-residues length peptide of sequence VPLPAGGGTVLTKMYPRGNHWAVGHLM that has a high affinity towards the BBR2 subtype [67]. BBR are neuroreceptors widely distributed among the nervous system and the gastrointestinal tract, playing a diverse role in several physiological and pathophysiological processes including the regulation of the circadian rhythm, feeding and obesity, neuronal disorders, chronic inflammatory diseases, and cancer (e.g., breast, prostate, lung, pancreas, and neuroendocrine, among others) [67]. BBR have been widely explored as drug targets for several antitumoral approaches, including targeted cancer diagnostics, precision therapy, and the selective delivery of antineoplastic agents, although the specific delivery of cytotoxic ruthenium and gold complexes into tumors by targeting the BBR has been barely explored and relies mainly on the use of bombesin-like peptides as delivering carriers [67].

**Figure 6:**
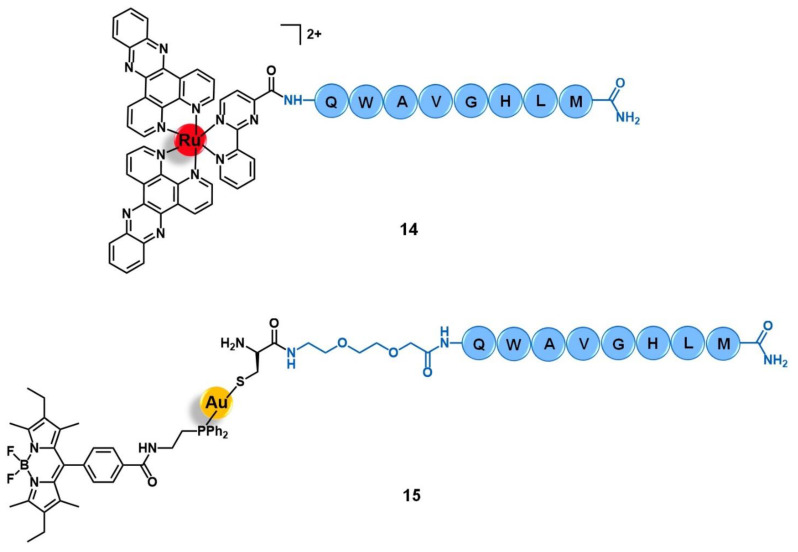
Ru(II) and Au(I) conjugates with peptides that target the bombesin receptors (BBR).

Aiming to improve the selectivity and efficacy of the cytotoxic complex [Ru(dppz)_2_(CppH)]^2+^ (dppz = dipyrido [3,2-*a*:2′,3′-*c*]phenazine; CppH = 2-(2′-pyridyl)pyrimidine-4-carboxylic acid) against human cervical cancer, Gasser et al. reported its conjugation to the truncated (7–14) form of bombesin QWAVGHLM (**14**, Figure 6) [68]. This sequence corresponds to the minimum required for binding to the BBR with high affinity. Given the overexpression of BBR2 in the HeLa cervical cancer cell line, the conjugation of the ruthenium complex to this peptide was expected to improve its selective internalization. The non-conjugated complex [Ru(dppz)_2_(CppH)]^2+^ displayed high cytotoxicity in HeLa cells (IC_50_ = 10.0 µM) and was shown to induce cell death by mitochondria-mediated apoptosis after being accumulated mainly in this cell organelle and presented an intrinsic low selectivity index (1.5) as compared to the MRC-5 non-tumoral lung fibroblast cell line (IC_50_ = 15.1 µM). Peptide conjugation resulted in an increase of cytoplasmic uptake, with a different pattern of accumulation, mainly along the nucleus region.

In addition, conjugate **14** showed a seven-fold decrease of cytotoxicity when compared to its precursor (IC_50_ HeLa = 71.8 µM; IC_50_ MRC-5 > 100 µM). These results suggest that despite receptor-targeting delivering peptides possibly increasing cargo uptake, the overall changes in the physicochemical properties (such as lipophilicity, charge, and size) might compromise the original cytotoxic activity by modifying the ability of the cargo to reach its pharmacological target [68].

Bodio and collaborators developed a potential theranostic Au(I) agent for precision anticancer therapy based on the fluorescent gold complex [Au(DPPEB-BODIPY)Cl] (DPPEB-BODIPY = *N*-[2-(diphenylphosphino)ethyl]-4-(1,3,5,7-tetramethyl-2,6-diethyl-4,4-difluoro-4-bora-3, 4a-diaza-sindacene-8-yl)benzamide) conjugated to a bombesin peptide derivative (**15**, Figure 6) [69]. The vectorizing peptide consists of the truncated (7–14) form of bombesin further derivatized with a PEG(2) spacer, which confers hydrophilicity to the conjugate and prevents the complex from interfering with the peptide binding ability, and a cysteine residue to allow the coordination to the metal center through the sulfur atom of its side chain. The conjugation considerably changed the properties of both the complex and the peptide moieties. Conjugate **15** showed only 21% fluorescence quantum yield while the non-vectorized complex [Au(DPPEB-BODIPY)Cl] showed 98%. However, even though the introduction of the peptide reduced the intense luminescence properties of the complex, the final conjugate still showed sufficient brightness for in vitro tracking. Moreover, the affinity of **15** to the bombesin receptors (BBR1 to BBR3) determined on rat cerebral cortex membranes was five-fold lower than of the reference full-length (1–14) bombesin peptide. Despite the reduction, conjugate **15** still showed a very high affinity on the nanomolar range (IC_50_ of displacement = 1.53 nM). The Au-peptide conjugate was 1.5-fold less cytotoxic than the precursor complex against PC-3 prostate cancer cells (IC_50_ = 115.9 µM and 74.9 µM, respectively), but 4.5-fold more cytotoxic in MDA-MD-231 breast cancer cells known to overexpress the BBR (IC_50_ = 10.9 µM and 25.1 µM, respectively). When comparing the cytotoxicity in MDA-MD-231 cells with that in non-tumoral HMEC human mammary epithelial cells, conjugate **15** showed a higher selectivity index than the non-vectorized complex (selectivity index MDA-MD-231/HMEC = 5.3 and 3.4, respectively). Interestingly, the cell uptake of both compounds is quite similar in the prostate and breast cancer cell lines, the compounds being distributed through the cytoplasm without entering the cell nucleus. In these cells, conjugate **15** was also shown to be brighter than the complex at a concentration range used for the IC_50_ determination. Given the results, the authors pointed to **15** as a selective anticancer agent capable of distinguishing between cancer cells overexpressing the BBR receptors versus those that do not overexpress it [69].

### 3.3. Opioid Receptors (OPR)

Opioid receptors are another class of GPCR that has been explored as targets for the selective delivery of ruthenium and gold complexes into different types of tumors. This class of neuroreceptors is typically expressed by neurons from both the central and the peripherical nervous systems, as well as by neuroendocrine, immune, and ectodermal cells [70,71]. They are subclassified into three different classes, namely δ-, κ-, and μ-opioid receptors, also known as OPR-1 to OPR-3, respectively. The designation of these receptors comes from the endogenous peptide ligands such as enkephalins, β-endorphin, and dynorphins, whose effects resemble opioid drugs [70]. Among the many roles of OPR in organism homeostasis and disease, these receptors are associated with the regulation of cell membrane potential, cell proliferation, emotional response, immune function, respiratory and cardiovascular systems, inflammation, pain, and neuronal transmission [70,71]. Many drugs targeting the OPR have been discovered and translated into clinical practice, most of them with an analgesic function [70]. The discovery of the association of OPR with cancer development and progression processes brought an increased interest from many research groups on these receptors and opened the possibility to exploring a new anticancer targeted approach [71]. In particular, the fact that OPR are overexpressed in certain types of adenocarcinomas, such as hormone-dependent and hormone-independent breast cancer, colorectal cancer, bladder cancer, and lung cancer, brings the opportunity to target these receptors with drug delivery systems for applications in precision medicine [71].

**Figure 7:**
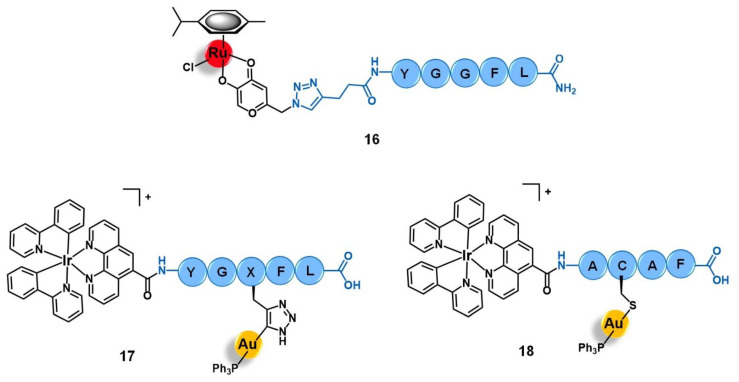
Ru(II)- and Au(I)-conjugates containing an enkephalin analogue (**16** and **17**) or a specific short-peptide (**18**) for targeting the opioid receptors (OPR). Note: X = propyrgyl-glycine.

Among the carriers used, enkephalin (a natural pentapeptide with two isoforms, the met-enkephalin YGGFM, and the leu-enkephalin YGGFL) and its derivatives are the most popular. Hartinger’s group reported the conjugation of complex [Ru(η^6^-*p*-cym)(azapyr)Cl] (azapyr = 2-(Azidomethyl)-5-oxo-4H-pyronate) to a leu-enkephalin peptide modified on the N-terminus with an alkyne group, by CuAAC click chemistry (**16**, Figure 7) [72]. Conjugate **16** was 13-fold more cytotoxic against CH1 human ovarian carcinoma cells than the respective non-conjugated Ru complex (IC_50_ = 13 µM and 168 µM, respectively), while the targeting peptide is not active per se (IC_50_ > 640 µM). Moreover, **16** is only chemosensitive to CH1 cells, since it was not active on SW480 colon adenocarcinoma and A549 non-small-cell lung cancer cell lines. In contrast, the non-conjugated complex was not able to distinguish between the three types of cancer cells. Interestingly, in solution, conjugate **16** hydrolyzes, giving an active species in which the chloride ion was exchanged for a water molecule. This activated conjugate can further react with certain nucleobases and amino acids, such as guanidine, histidine, cysteine, or glycine. These reactions are characterized by ligand exchange with the pyronate-peptide moiety, which releases the vectorizing peptide and yields a complex of general formula [Ru(η^6^-*p*-cym)(biomolecule)]^+^ (the biomolecule corresponds to the stated nucleobase and/or amino acids). This behavior, whose interaction was preferable and stronger with the amino acids rather than with the nucleobases, suggests that the Ru-peptide conjugate and the non-vectorized complex might have a cellular target different from the DNA and more likely related to a protein. Altogether, these results suggest that the conjugation of leu-enkephalin to this ruthenium complex is reversible, which allows the release of the active moiety to the cancer cells and thus prompts both its activity and selectivity [72].

Gimeno and co-workers developed a bimetallic gold(I)/iridium(III)-enkephalin conjugate (**17**, Figure 7) for potential application as a theranostic agent for the targeted therapy and imaging of lung cancer [73]. This conjugate was prepared firstly by conjugation of the precursor complex [Ir(ppy)_2_(phenCOONa)] (ppy = 2-phenylpyridine; phenCOONa = sodium 1,10-phenanthroline-5-carboxylate) to a leu-enkephalin analogue derivatized with a propargyl group of sequence YGXFL (X = propyrgyl-glycine); followed by the click reaction of this intermediate conjugate with [AuN_3_PPh_3_]. The rationale behind the design of **17** was based on bringing together three moieties for complementary purposes: a fluorophore Ir(III) complex for imaging; a bioactive Au(I) complex for therapy; and an enkephalin-like peptide for cancer cells targeting and transport of both complexes while also conferring water solubility to the conjugate. Conjugate **17** showed excellent luminescence properties for fluorescence cell microscopy with around 21% quantum yield, mainly arising from the iridium complex whose emission spectra suffered a red shift towards approximately 615 nm after peptide conjugation. The conjugate did not show any antiproliferative activity against A549 non-small-cell lung cancer cells (IC_50_ > 50 µM) despite its uptake, where the conjugate was essentially localized in the cytoplasmic area close to the nucleus without penetrating it nor the mitochondria. This evidence suggests an entrapment of conjugate **17** in the lysosomes, with very slow escape, which in turn prevents it from interacting with its potential pharmacological target, namely the mitochondria as this is a known common target of both iridium and gold complexes. Interestingly, a different behavior was found for an analogue conjugate (**18**, Figure 7) reported by the authors, in which the propargyl functionalized leu-enkephalin carrier was replaced by a short peptide of sequence ACAF [73]. This sequence was chosen to explore an alternative conjugation approach, by including a cysteine residue whose sulfur at the side chain would allow direct coordination to the gold moiety. Although **18** showed an analogous lysosome-mediated cellular uptake by A549 cells, this conjugate was revealed to be more cytotoxic (IC_50_ > 12.3 µM) than its analogue. The different overall charges of the conjugates (**17** is cationic while **18** is neutral) influences their ability to escape from the lysosomes as the neutral conjugate escaped better. On the other hand, the different coordination sphere of the gold complexes (Au-C bound in **17** versus Au-S bond in **18**) might also play a crucial role in the releasing of the bioactive moiety from the delivery peptide to interact with its target further freely, as the AU-S bond tend to be more easily cleaved in physiological conditions. According to the authors, these two factors could be a possible explanation for the differences observed in the cytotoxicity of the two conjugates, giving an insight into some features that could influence the correct delivery of gold complexes into cancer cells by using specific targeting peptides [73]. Thus, this work highlights the importance of appropriate cellular uptake of the complexes of interest and that is achieved not only based on the nature of the targeting peptide but also due to the properties of the cargo itself.

### 3.4. G Protein-Coupled Estrogen Receptors (GPER)

Estrogen receptors (ER) comprise two distinct classes of proteins, classical versus G-protein coupled, that act as receptors of the endogenous estrogen hormones, playing a key role in the function and maintenance of the reproductive, endocrine, cardiovascular, immune, and nervous systems [74,75]. The classical ER, which can be subdivided in ER-α and ER-β, are intracellular receptors expressed in the nuclear membrane, thus being part of the nuclear hormone receptors superfamily, while the G protein-coupled estrogen receptors (GPER) are transmembrane protein members of the GPCR superfamily [74]. The mechanism of action and effects of the two ER classes upon activation by estrogen differ substantially: the classical receptors are ligand-activated transcription factors that can translocate into the cell nucleus, bind to DNA, and regulate gene expression, whereas GPER mediate the transduction of extracellular signals through the activation of intracellular cascade processes mediated by the G protein [74,75]. Disfunction and alterations of ER are commonly associated with many diverse pathophysiological conditions, including cancer [75,76]. The development and growth of many types of tumors, such as breast and ovarian, show well-established ER-dependent mechanisms and therefore they have been intensively studied as drug targets for anticancer therapy and imaging. Many drugs clinically available for the treatment of hormone-dependent breast cancers target these receptors, and an increasing number of novel drug candidates are currently under development [75,76]. Additionally, these receptors also hold an important role as cancer biomarkers and prognostic tools, which further motivates the development of ER-targeting agents [61].

Given the important implacability of ER in cancer, some authors have also exploited the use of ER-targeting agents as carriers for the selective delivery of cytotoxic ruthenium and gold complexes into cancer cells for potential application in precision medicine. These approaches make use of estrogen-like ligands, mainly derivatives of 17β-estradiol, with high affinity towards the ER, selective modulators of these receptors (such as tamoxifen), and inhibitors of aromatase as this is an enzyme involved in the synthesis of estrogen (e.g., anastrozole and letrozole).

**Figure 8:**
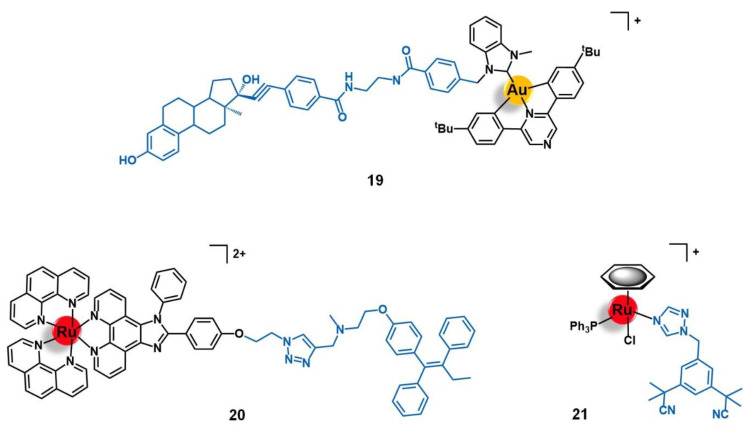
Au(III) and Ru(II) complexes containing an estradiol-derivative (**19**), tamoxifen (**20**), or anastrozole (**21**) for the targeting of the protein-coupled estrogen receptor (GPER). Note: ^t^Bu = *tert*-butyl.

Bertrand and co-workers reported the conjugation of the pyrazine-based pincer gold(III) complex [Au(bbfpz)(acbim)]^+^(bbfpz = 2,6-bis(4-(*tert*-butyl)phenyl)pyrazine; acbim = 1-methyl-3-(4-(6-aminohexyl)carboxamido)benzylbenzimidazol-2-ylidene) to a derivative of 17α-ethinylestradiol (**19**, Figure 8), aiming to increase its selectivity towards ER(+) breast cancer cells [77]. The authors also reported two other analogues, where the gold complex was separated from the targeting moiety by shorter linkers (2 and 4 carbons instead of 6 carbons). They showed that the complexes become more active against MCF-7 breast cancer cells as the linker’s length increases, which is in good agreement with the need for an appropriate distance between both functionalities to prevent stereo blockage. Conjugate **19** showed slightly higher cytotoxic activity against ER(+) breast cancer cells (MCF-7) than ER(−) ones (MDA-MB-231) and also higher than a non-tumoral fibroblast cell line (MRC-5), with IC_50_ values of 5.9 µM, 9.3 µM, and 9.1 µM respectively. Moreover, the cellular uptake of **19** was equally significantly higher in the MCF-7 (ER+) cells compared to the MDA-MB-231 (ER−) line, which supports the antiproliferative results as well as suggesting a possible targeting effect given by the 17α-ethinylestradiol. However, **19** was shown to be up to 10-fold less active in these cell lines once comparing to the free gold complex that was not conjugated to the ER-targeting vector, despite its higher level of internalization. These apparently contradictory findings could be eventually explained by the decreased ability of the conjugate to interact with G-quadruplex DNA structures observed. The authors thus suggested that detachable vectors that allow the releasing of the active complex to freely interact with its target is essential so that the increase of selectivity would not be impaired by the decrease of activity [77].

The first ER-targeted Ru(II) polypyridyl complex designed for two-photon PDT of breast cancer cells (**20**, Figure 8) was reported by Fan and co-workers [78]. The luminescent complex [Ru(phen)_2_(phenimi)]^2+^ was conjugated by CuAAC click chemistry to tamoxifen, which is one of the most commonly used drugs for the antiestrogenic treatment of breast cancer. Here, tamoxifen acts as an ER-specific targeting moiety which is connected to the Ru photosensitizer by a triazole-containing linker. This conjugate showed similar photochemical properties and quantum yields as those of the respective precursor ruthenium complex, with intense absorption at approximately 460 nm and fluorescence at 600 nm. The cellular uptake of **20** by ER(+) MCF-7 cells was significantly higher than that of the non-conjugated ruthenium complex. Additionally, **20** had little appreciable internalization by the ER(−) MDA-MB-231 breast cancer cells, and by the non-tumoral cell lines HL-7702 (human liver cells) and COS-7 (kidney fibroblasts). Under two-photon irradiation (830 nm), **20** showed higher cytotoxicity than the non-vectorized complex against MCF-7 cells, inducing cell-death via lysosome-disruption upon generation of ^1^O_2_. Given the promising results, the authors claim the potential of conjugate **20** to be further developed as a specific PDT anticancer agents for ER(+) breast cancers [78].

A different approach for delivering organometallic ruthenium complexes into breast cancer cells by introducing known-aromatase inhibitors as co-ligands was reported by Castonguay et al. [79]. These authors studied a series of Ru(II)-arene complexes bearing the third-generation aromatase inhibitor anastrozole, among which the complex [Ru(η^6^-C_6_H_6_)(PPh_3_)(η^1^-ATZ)Cl]^+^ (ATZ = anastrozole, **21**, Figure 8) was the most promising. The incorporation of ATZ into the complex did not impair its ability to interact with aromatase. Indeed, **21** was able to decrease its activity in H295R adrenocortical carcinoma cells (86% aromatase inhibition at 1 µM of **21**), which was in good agreement with the docking simulations performed for the complex with a human placental aromatase cytochrome P450 (CYP19A1) model. Additionally, **21** showed high cytotoxicity against two ER(+) breast cancer cell lines (MCF-7 and T47D) with IC_50_ values of ca. 4 µM, while the free ATZ was not active, suggesting that the ruthenium moiety is responsible for the antiproliferative activity observed. A high level of cell uptake (> 30 ng/10^6^ cells) was also observed for the complex in MCF-7 cells, supporting the previous results found. In zebrafish embryos, **21** (at 12.5 µM) did not lead to noticeable signs of toxicity over 96 h, which together with the high antiproliferative and anti-aromatase activities prompted the authors to consider this complex a suitable candidate for further investigation as a potential anticancer agent against ER(+) breast cancer [79].

## 4. Growth Factors Receptors (GFR)

Growth factor receptors (GFR) are a highly diverse superfamily of cell membrane proteins that act as receptors of their cognate ligands, the growth factors [80]. These ligands, as the name suggests, are endogenous molecules to biological organisms, such as hormones (protein or steroidal) and cytokines, capable of inducing and regulating cell proliferation, tissue growth, differentiation, and healing (e.g., insulin, interleukins, erythropoietin, thrombopoietin, epidermal growth factor, fibroblast growth factor, and tumor necrosis factor, among many others) [80]. Etymologically, GFR are classified according to the respective growth factor, and the great majority of them are receptor tyrosine kinases (RTK) [80]. The latest are transmembrane proteins involved in the most diverse of the physiological processes, characterized by an extracellular binding-domain and an intracellular kinase domain responsible for catalyzing the transfer of phosphate from ATP molecules to the tyrosine residues in peptides and proteins, promoting the regulation of the biological processes where they are involved [81]. There are 20 different classes of RTK, not all of them being GFR as well. The disfunction of these receptors is associated with several diseases including cancer, for which intensive research has been undertaken and several drugs have been discovered. The overexpression of these receptors in many cancer types also opened the door for precision medicine approaches and targeted drug delivery [80,81,82]. In the case of targeted delivery of ruthenium and gold complexes into cancer cells, the reported studies targeting the GFR mainly focus on the epidermal growth factor receptor (EGFR), human epidermal growth factor receptor 2 (HER2), and fibroblast growth factor receptor (FGFR), all three being RTK as well. RTK class I family comprises all the receptors of the epidermal growth factor, including four different sub receptors (EerB-1 to EerB-4) of which EGFR corresponds to EerB-1 while HER2 is EerB-2. The FGFR corresponds to RTK class V [81].

### 4.1. Epidermal Growth Factor Receptor (EGFR)

The EGFR signaling network plays a primary role in the growth, maintenance, and homeostasis of epithelial tissues [82,83]. Many different types of cancer (e.g., lung, colorectal, pancreatic, breast, ovarian, cervical, bladder, neuroendocrine, glioma head, and neck) are associated with abnormalities in the EGFR axis, either by receptor overexpression, disfunction due to mutations, and/or increased autocrine and paracrine production of its growth factors [82,83]. Consequently, a huge number of anticancer drugs targeting EGFR are under clinical use or development, an approach that became a prime example of personalized targeted medicine [82]. Furthermore, EGFR can mediate the endocytosis of external agents into cancer cells, as well as translocate to the nucleus and the mitochondria, for which these receptors have been intensively studied as targets for the delivery of several chemotherapeutic, radionuclides, immunotoxins, and photosensitizers [83].

Several groups have been developing EGFR-targeting delivery systems for cytotoxic inorganic and organometallic ruthenium and gold complexes, whose approaches are based either on the conjugation of an EGFR ligand to the complexes or on its direct coordination to the metal center.

**Figure 9:**
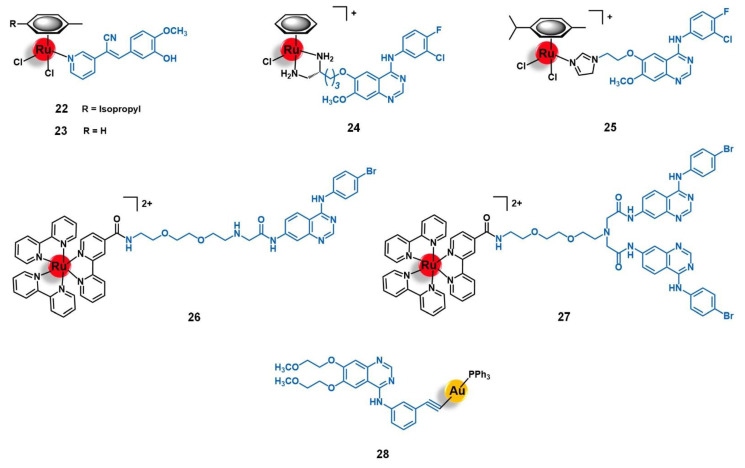
Ru(II) and Au(I) complexes that target the epidermal growth factor receptor (EGFR). The vectorizing agents include tyrphostin-like small molecules (**22** and **23**), derivatives of 4-anilinoquinazoline (**24** to **27**), and erlotinib (**28**).

Schobert and colleagues reported two new Ru(II)-arene complexes containing a tyrphostin-like ligand, [Ru(η^6^-*p*-cym)(TYR)Cl_2_] and [Ru(η^6^-toluene)(TYR)Cl_2_] (**22** and **23**, respectively, Figure 9), in which TYR = 3-(1’-cyano-2’-(3’’-hydroxy-4’’-methoxyphenyl)(*Z*)-ethenyl)pyridine [84]. Tyrphostins are a known class of EGFR inhibitors, and therefore the rationale behind the design of this study was based on studying to which extent it would be possible to obtain synergistic effects from bringing together the EGFR-targeting moiety and the cytotoxic ruthenium core. The compounds were screened against a panel of EGFR(+) (MCF-7/topo multidrug resistant breast adenocarcinoma) and EGFR(−) (518A2 melanoma, HL-60 leukemia, and KB-V1/Vbl cervix cancer) cancer cell lines. Both complexes showed high cellular internalization by 518A2 cells, and interacted strongly with DNA without, however, altering its topology. Interestingly, the two complexes were shown to be significantly more active against all cell lines than the free tyrphostin ligand, with **23**, which showed a surprisingly decrease of activity in the EGFR(+) breast cancer cells, as an exception. While **22** was particularly more cytotoxic against MCF-7/topo cells, most probably due to EGFR-targeting, complex **23** demonstrated a specific anti-melanoma activity, possibly dependent on the Akt/mTOR signaling pathway. Brought together, these results gave new insights into how modification in the metal complexes might modify the targeting ability of the vector agent, thus redirecting it towards alternative biological targets [84].

Wang and co-workers conducted extensive work on the development and biological evaluation of several Ru(II)-arene organometallic complexes with ligands derived from the EGFR-inhibiting agent 4-anilinoquinazoline (AQZ) for targeted anticancer therapy [85,86,87]. The 4-anilinoquinazoline family includes the well-known anti-EGFR drug Gefitinib, which was the first of its class to obtain a marketing authorization. The extensive number of complexes synthesized by Wang’s group with different arenes (e.g., benzene, *para*-cymene, biphenyl, indane, and 2-phenylethanol) and several AQZ derivatives gave a broad knowledge of the structure-anticancer activity and the structure-EGFR affinity relationships of this type of complex. In a general way, the authors found that coordination of the ruthenium core with the AQZ-like ligands preserved both the ability of the metal center to interact with the DNA upon hydrolysis, via minor groove binding and nucleobase coordination, and the ability of the ligand to recognize its receptor with high affinity (nM range). Additionally, a synergistic effect between both moieties was observed for the complexes, which showed enhancing pro-apoptotic activities (particularly early-stage apoptosis) in different EGFR(+) cancer cells lines (MCF-7 breast adenocarcinoma, HeLa cervical tumor, and A549 non-small-cell lung cancer). As expected, the majority of the complexes were shown to be more active against EGFR-stimulated growth cell lines of several types of human tumors (breast, cervix, lung, prostate, and skin) [85,86,87]. Among the compounds evaluated, two lead compounds were identified, namely [Ru(η^6^-benzene)(enAQZ)Cl]^+^ (**24**, Figure 9, enAQZ = 4-(3′-chloro-4′-fluoroanilino)-6-(2-(2-aminoethyl) aminoethoxy)-7-methoxyquinazoline) and [Ru(η^6^-*p*-cym)(enAQZ)Cl]^+^ (**25**, Figure 9, enAQZ = 6-(2-(2-(1H-imidazol-1-yl))ethoxy)-4-(3’-chloro-4’-fluoroanilino)-7-methoxy-quinazoline). Complex **24** (IC_50_ to EGFR = 29.1 nM) was revealed to be a promising antiproliferative agent against MCF-7 breast (IC_50_ = 17.3 µM) and HeLa cervical cancer cells (IC_50_ = 1.4 µM), with selectivity index values (EGFR-induced versus non-induced growth of the cell lines) higher than 6 and 3, respectively [85,86]. Complex **25** (IC_50_ to EGFR = 66 nM) showed high cytotoxicity against the A549 non-small-cell lung cancer cell line (IC_50_ = 15 µM), as well as a tendency to bind preferably to the cell membrane (where EGFR is located) with a portion entering the cell to exert a dual-effect on both enzyme inhibition and DNA binding [87]. Altogether, these results suggest that ruthenium complexes with AQZ-like anti-EGFR ligands hold great potential to be further developed as dual-mode anticancer agents for targeted therapy.

The potential of AQZ derivatives for the delivery of ruthenium complexes into cancer cells mediated by the EGFR receptors was also explored by Georgiades et al. [88]. The authors developed a targeted theranostic agent for cancer applications, by conjugating the complex [Ru(bipy)_2_(bipyCOOH)]^2+^ (bipyCOOH = 2,2′-bipyridine-4-carboxylic acid) to the EGFR-inhibitor AcetAQZ (*N*-(4-((4-bromophenyl)amino)quinazolin-7-yl)-2-chloroacetamide), through a triethyleneglycol-derived diamino linker, affording the mono-conjugate **26** and the bis-conjugate **27** (Figure 9). The structure of the conjugates was designed following the rationale of including a fluorophore moiety based on (i) the well-known luminescent complex [Ru(bipy)_3_]^2+^, (ii) tethered to the EGFR-targeting vector AcetAQZ that was selected upon the synthesis and evaluation of an anilinoquinazoline library screened against SW480 grade II colon cancer cells expressing a mutant form of EGFR, and (iii) using a triethyleneglycol-like spacer between both moieties not only to keep the fluorophore farther away for the receptor’s kinase hinge region but also to enhance the aqueous solubility of the conjugate. Both conjugates revealed up to 2.5-fold higher cytotoxicity against EGFR-mutant SW480 cells than the precursor complex [Ru(bipy)_2_(bipyCOOH)]^2+^, **27** being the more active of the two, most likely due to the higher overall positive charge, which may enhance cell membrane permeability and uptake by negatively charged organelles. Indeed, this conjugate was efficiently taken up by the cells, with a specific mitochondrial localization potentially associated with the mitochondria-translocated forms of EGFR. These results prompted the authors to propose conjugate **27** as a potential platform for targeting translocalized mutant forms of EGFR and for the delivery of theranostic agents into cancer cells or across the blood-brain barrier (BBB) given the strong negative charge of tumoral cells and the BBB endothelial cells [88].

The vectorization of gold(I) complexes toward tumors by targeting the EGFR receptor was barely explored. Ruiz and co-workers developed complex [Au(erlotinib)(PPh_3_)] (**28**, Figure 9) using erlotinib, a well-known EGFR inhibitor clinically employed as an anticancer drug for the treatment of patients with non-small cell lung cancer or pancreatic tumors, as an EGFR-targeting ligand [89]. The authors explored the eventual synergistic effects from bringing together the [Au(PPh_3_)]^+^ fragment, known for its cytotoxicity, and the EGFR-targeting drug. Complex **28** showed higher cytotoxicity than the free erlotinib against MCF-7 and MDA-MB-231 breast and HT-29 colon cancer cell lines with IC_50_ values on the low micromolar range, with particularly efficacy against EGFR(+) MDA-MD-231 cells (IC_50_ = 1.6 µM) in which it showed a 68-fold increase of activity. Additionally, the complex was also shown to selectively target the cancer cell lines compared to the BGM non-tumorigenic kidney cells, with selectivity index values up to 10. In the triple-negative MDA-MB-231 breast cancer cells, **28** showed a mechanism of action involving mitochondrial disfunction, DNA damage, and production of ROS, which led to cell cycle arrest at S and G_2_/M phases, and eventual cell death by apoptosis. Compared to the free erlotinib that causes cell arrest at the G_1_/S transition, the introduction of the gold moiety promoted a drastic modification of its bioactivity while keeping its selectivity towards EGFR-expressing cancer cells. Thus, the authors state that **28** showed promising features to be further developed as an anticancer agent for targeted medicine [89].

### 4.2. Human Epidermal Growth Factor Receptor 2 (HER2)

Despite being less explored than the EGFR, the HER2 has been under the spotlight of personalized anticancer therapy as well [90]. This receptor is the pharmacological target of a class of drugs that is very well established in the clinical practice of patients with HER2(+) breast adenocarcinoma and HER2(+) gastric cancer. Given the overexpression or aberration of this receptor in many other solid tumors, such as colorectal, non-small-cell lung, biliary tract, and bladder cancers, several HER2(+)-targeting anticancer drug candidates are currently under clinical trials [90]. HER2 has also been intensively explored as a target for the selective drug delivery of antineoplastic agents into tumors overexpressing it [90].

**Figure 10:**
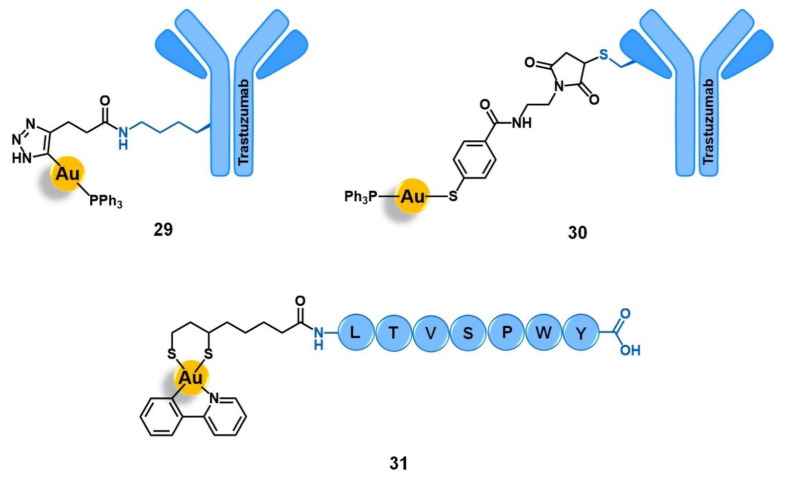
Conjugates of Au(I) with the monoclonal antibody Trastuzumab (**29** and **30**) or Au(III) with the peptide LTVSPWY (**31**) for targeting the human epidermal growth factor receptor 2 (HER2).

In this frame, the studies reporting the vectorization of gold(I) and gold(III) complexes into HER2(+) tumoral cells for potential application in breast cancer therapy are based on the use of antibodies or peptides with high affinity and selectivity towards HER2 as vectors. Contel et al. reported two new antiproliferative gold(I)-antibody conjugates based on the Trastuzumab drug (**29** and **30**, Figure 10) [91]. Trastuzumab, also known as herceptin, is an anti-HER2 humanised IgG1 monoclonal antibody approved for the treatment of HER2(+) breast, metastatic gastric, and gastroesophageal cancers either used in monotherapy regimens or in combination with other anticancer drugs. Compound **29** was prepared by conjugating the cytotoxic complex [Au(PPh_3_)(DPTP)] (DPTP = 2,5-dioxopyrrolidinyl-3-(1*H*-1,2,3-triazol-4-yl)propanoate) to Trastuzumab via reaction of the *N*-hydroxysuccinimide moiety of the complex with the lysin residues of the antibody (non-site specific modification). Compound **30** was obtained by conjugating Trastuzumab to the complex [Au(PPh_3_)(MBANHS)] (MBANHS = 4-mercaptobenzylmaleimido propionamide) through the reaction of its maleimide group with the cysteines available at the antibody (site-specific approach). The conjugation of the gold complexes to Trastuzumab maintained the high affinity of the antibody towards HER2 (EC_50_ Trastuzumab = 0.22 nM; EC_50_ **29** = 1.13 nM; EC_50_ **30** = 0.36 nM). Both conjugates are significantly more cytotoxic than the non-conjugated complexes and the free antibody, with EC_50_ values in the sub-micromolar range against the HER2(+) MCF-7 and BT-474 breast cancer cells. Moreover, conjugates **29** and **30** are also more active against the HER2(+) cell lines than against the HER2(−) cells, demonstrating a higher degree of selectivity than the respective parent complexes with index values up to 12. The authors suggested that the higher affinity, cytotoxicity, and selectivity presented by conjugate **30** compared to **29** could be assigned to the non-site-specific modification of the antibody through the lysine-conjugation approach on **29**. Nevertheless, both compounds showed encouraging results and have the potential to be further explored for the targeted anticancer therapy of HER2(+) breast cancer [91]. From our point of view, the use of antibodies, like Trastuzumab, seems to be a promising approach to target the HER2, which can be expanded to other metals, namely to ruthenium complexes.

Metzler-Nolte and co-workers also reported the conjugation of the gold(III) complex [Au(ppy)(Lpa)] to peptide LTVSPWY, which is known to facilitate the uptake of cargos into breast cancer cells through HER2 targeting (**31**, Figure 10) [52]. This conjugate was shown to be two-fold and six-fold more active than the precursor complex [Au(ppy)Cl_2_] in MCF-7 and MDA-MD-231 cancer cell lines, respectively. Despite the improvement in the antiproliferative activity observed, probably due to higher cell uptake mediated by HER2, **31** was still less active than the analogous conjugate **7** (Figure 1) with the integrin-targeting *cyclo*-RGDfK peptide [52]. Nonetheless, these results are still very encouraging to further explore the HER2-targeted pathway for the selective delivery of gold complexes into breast cancer cells.

### 4.3. Fibroblast Growth Factor Receptor (FGFR)

Fibroblast growth factor receptors are composed of four different subclasses (FGFR1 to FGFR4) with over 22 endogenous growth factors, involved in diverse physiological processes such as embryogenesis, organogenesis, angiogenesis, and tissue growth, differentiation, and repair [92]. Many congenital disorders and cancers are associated with FGFR dysfunction and/or overexpression. The FGFR pathway was identified to play a critical role in cancer cell proliferation and invasion, resistance to anticancer therapy, and neoangiogenesis [92,93]. Many current antineoplastic drugs target FGFR, although in a non-selective way and having several other pharmacological targets, an increasing number of selective agents (FGFR agonists and antagonists) are under development and clinical evaluation [93]. The different FGFR subtypes are also associated with different cancer types, including breast, ovarian, lung, bladder, gastric, endometrial, and skin [93]. Some of the stated tumors are associated with more than one subreceptor, such as the case of breast cancer in which around 20% of the cases overexpress at least one of the FGFR1 to FGFR3 receptors. The presence of FGFR alterations in breast cancer is also associated with poor prognosis, higher metastatic potential, early disease relapse, and failure of the conventional anticancer therapy [94].

**Figure 11:**
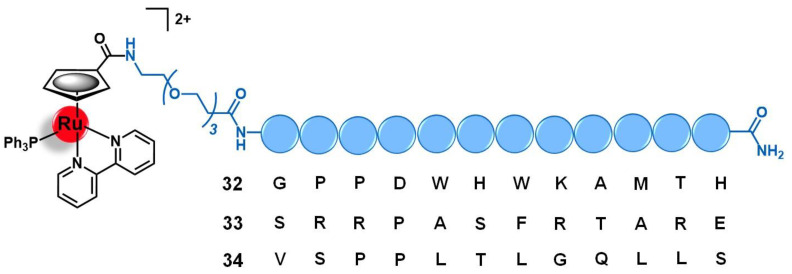
Ru(II)-conjugates for targeting the fibroblast growth factor receptor (FGFR) containing the peptides GPPDWHWKAMTH (32), SRRPASFRTARE (33), or VSPPLTLGQLLS (34) as vectors.

Aiming to develop target-specific anticancer agents for metastatic breast cancer, Morais and co-workers recently reported the conjugation of a cytotoxic ruthenium(II) organometallic complex to FGFR-targeting peptides [95]. The rationale underlying this approach was based on selectivity improvement of the known cytotoxic complex [Ru(Cp)(bipy)(PPh_3_)]^2+^, by using peptide vectors that could selectively target the FGFR(+) breast cancer cells while sparing the non-tumoral tissues that have an intrinsic lower expression of FGFR. Firstly, the authors performed molecular dynamic studies with the ruthenium complex in a cell membrane model to evaluate which position of the complex would be more suitable for derivatization and peptide conjugation without affecting its ability to interact with the cell membrane, a key process for its activity. After identifying the Cp ligand as one of the most favorable positions, the authors derivatized it with a carboxylic acid group for amide coupling, obtaining the complex [Ru(CpCOOH)(bipy)(PPh_3_)]^2+^ (where CpCOOH = cyclopentadienylcarboxylic acid). Three peptides with high affinity to FGFR1 (GPPDWHWKAMTH), FGFR2 (SRRPASFRTARE), or FGFR3 (VSPPLTLGQLLS) were selected as targeting moieties and were conjugated to [Ru(CpCOOH)(bipy)(PPh3)]^2+^ through a PEG(3) spacer (**32** to **34**, respectively; Figure 11). The spacer not only improves water solubility, but also allows the cytotoxic agent and the delivering vector to be far apart so that the activity and affinity of each one are not affected by the other. All conjugates showed to be less active than the precursor complex [Ru(CpCOOH)(bipy)(PPh_3_)]^2+^ against FGFR(+) SKBR3 and FGFR(−) MDAMB231 breast cancer cell lines, which in turn was also less cytotoxic than the parent complex [Ru(Cp)(bipy)(PPh_3_)]^2+^. Nevertheless, the three conjugates showed to be more cytotoxic against FGFR(+) SKBR3 cells than against FGFR(−) MDAMB23, particularly conjugate **34**. Indeed, the latter showed a difference in the cell viability between FGFR(+) and FGFR(−) lines higher than 25%, while the free ruthenium complexes were unable to distinguish between the two cell lines. Altogether, the results suggest that despite both the derivatization and peptide conjugation leading to a cytotoxicity loss of the parent complex, introducing FGFR-targeting peptides increased its selectivity towards FGFR(+) breast cancer cells. The authors postulated that this approach has the potential to be further developed and to give effective target-specific anticancer agents; however, there is a need to modify the method for chemical derivatization and peptide conjugation so that the original anticancer activity of the metal complex is kept [95].

Among the three classes of growth factors receptors herein discussed, the FGFR is the less exploited of these cell targets for the specific delivery of ruthenium and gold complexes into tumors. However, the preliminary results found for the ruthenium-peptide conjugates targeting the FGFR suggest that this strategy, once further optimized, holds great potential to one day become a new approach in the precision therapy of cancer.

Table 1 summarizes the ruthenium and gold complexes that were conjugated to different vectors exploited to target emerging molecular receptors, namely cell adhesion molecules (integrins and cadherins), G-protein coupled receptors (somatostatin receptors, bombesin receptors, and opioid receptors) and growth factor receptors (epidermal growth factor receptor, human epidermal growth factor receptor 2, and fibroblast growth factor receptor).

## 5. Other Emerging Targets

Apart from the three major classes discussed above, other molecular receptors have been targeted aiming to deliver ruthenium or gold complexes into tumoral cells. Among them, the non-GPCR steroid hormone receptors, such as the progesterone receptors (PR), have been emerging as promising alternatives to conventional targets [96,97] and will be further discussed with higher detail in the following subsection.

Although not reviewed in detail in this manuscript, biomolecules, such as amino acids, vitamins, and carbohydrates, are among the most common and broadly studied delivering agents of anticancer drugs into tumors [11]. There are numerous reports of bioconjugates containing ruthenium and gold complexes for targeted anticancer applications that have been reported and discussed elsewhere [98,99,100,101,102]. Herein, we selected a couple of examples for illustrative purposes (see Section 5.2).

Apart from the stated molecular targets, conjugates of ruthenium and gold complexes with delivering agents aiming for the cancer cell organelles (e.g., mitochondria, nucleus, membrane) have been studied as well, of which we will briefly discuss those containing organelle-specific vectors in the last subsection of this manuscript.

### 5.1. Progesterone Receptors (PR)

Progesterone receptors (PR) comprise two different groups of proteins that act as the endogenous receptors to the cognate hormone progesterone, classified according to their cellular location as nuclear (classical) or membrane (extra-nuclear alternative) PR receptors [103,104]. The nuclear PR belong to the 3-ketosteroid receptor subfamily of the nuclear steroid hormone receptors that, upon ligand binding, mediate genomic signaling after translocation to the DNA. The membrane PR are transmembrane receptors that mediate intracellular signaling cascades via a non-G-protein-dependent pathway [103]. Both classical and alternative PR signaling mechanisms are involved in the control of several physiological processes related to the developmental and reproduction processes, including the growth and differentiation of the reproductive system organs [103,104]. Several diseases are associated with PR disfunction, including cancer (e.g., breast, ovarian, and brain tumors) where they mediate cancer cells survival, proliferation, and metastasis [104,105]. The PR have a particularly well-studied key role in the progress of hormone-dependent breast cancers, with many anticancer PR-targeting drugs used for treating PR(+) breast cancers [105]. Given the overexpression of PR in some types of cancer cells, they have also been explored as targets for the precise drug delivery of antineoplastic agents into tumors. As regards ruthenium-based metallodrugs, some authors have reported the conjugation of organometallic Ru(II)-arene complexes with testosterone-like ligands for potential application in the targeted therapy of breast cancer.

**Figure 12:**
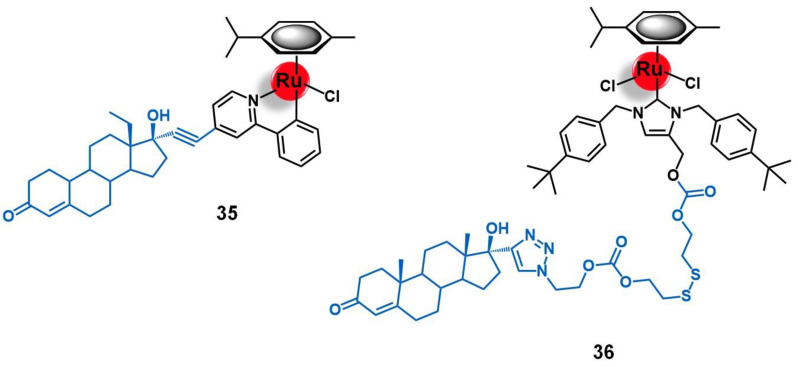
Ru(II)-conjugates that target the progesterone receptor (PR): 35 contains the steroid levonorgestrel as vector, while the **Table 36.** is the result of the conjugation of [Ru(η^6^-*p*-cym)(NHC)Cl_2_] complex to a testosterone-derived delivery agent through a glutathione-sensitive linker.

Ruiz and co-workers reported the synthesis of complex [Ru(η^6^-*p*-cym)(LEV-ppy)Cl] (**35**, Figure 12, LEV-ppy = 17-α-[2-phenylpyridyl-4- ethynyl]-19-nortestosterone) for the targeted therapy of PR(+) cancers [96]. The rationale of the design of this compound consisted of conjugation of the cytotoxic ruthenium complex [Ru(η^6^-*p*-cym)(ppy)Cl] to the steroid levonorgestrel (LEV), which displays high affinity towards the PR as the vector. Conjugate **35** showed high activity in the low micromolar range against PR(+)breast cancer cells (IC_50_ T47D = 7.4 µM) and epithelial ovarian carcinoma cells (IC_50_ A2780 = 3.7 µM; and IC_50_ A2780cisR = 3.1 µM). Comparatively, the non-conjugated complex [Ru(η^6^-*p*-cym)(ppy)Cl] was 13- to 23-fold less active, while free LEV was not active against any of the stated cell lines (IC_50_ > 100 µM). Additionally, **35** interacts with DNA upon ligand exchange and coordination to guanidine, altering the superhelicity degree of DNA. Altogether, the results suggest that the conjugation of ruthenium complexes to levonorgestrel increases its activity towards PR(+) cancer cells while keeping its ability to interact with the biological targets [96].

Aiming to enhance the therapeutic effect of the ruthenium-*N*-heterocyclic carbene [Ru(η^6^-*p*-cym)(NHC)Cl_2_] (NHC = (1,3-bis(4-(*tert*-butyl)benzyl)-2,3-dihydro-1*H*-imidazol-4-yl)methanol) against PR(+) breast cancer, Lin’s team reported its conjugation to 17-α-ethynyltestosterone through a disulphide-linker [97]. The resulting conjugate [Ru(η^6^-*p*-cym)(Te-SS-NHC)Cl_2_] (**36**, Figure 12; Te-SS-NHC = 17-α-[1,3-bis(4-(*tert*-butyl)benzyl)-4-methyl-2,3-dihydro-1*H*-imidazole-bis(2-diethylcarbonate)disulfide-1*H*-1,2,3-triazol-1-yl]-19-testosterone) was sensitive to glutathione (GSH), inducing the release of [Ru(η^6^-*p*-cym)(NHC)Cl_2_] from the vector. Thus, conjugate **36** can be considered a tumor stimuli-dependent pro-drug, given the high levels of GSH in the tumoral tissues. This conjugate displayed a higher cytotoxic activity against the PR(+) MCF-7 breast cancer cell line (IC_50_ = 4.5 µM) compared to the PR(−) MDA-MB-231 cells (IC_50_ = 20.7 µM, selectivity index = 4.5). On the contrary, the non-conjugated complex showed similar cytotoxicity in the two cell lines, being two-fold less active than conjugate **36** against the PR(+) cells. The difference in the antiproliferative activity can be explained by the higher degree of cell uptake of **36** by MCF-7 cells compared to [Ru(η^6^-*p*-cym)(NHC)Cl_2_]. Indeed, upon conjugation to the testosterone-derived vector, the uptake of **36** is highly enhanced in the PR(+) cells, while it remains almost the same in the PR(−) cell line. The authors also showed that the reduction of viability of the tumoral cells by the conjugate is due to cell cycle arrest at the S and G2/M phases combined with the induction of apoptosis, which is stronger in the MCF-7 cells rather than in the MDA-MD-231 cell line. Additionally, in an MCF-7 xenograft model of nude mice, conjugate **36** showed a higher level of accumulation in the tumor and a lower level of retention in the liver compared to the non-conjugated complex, which resulted in smaller tumor volume and a longer mice survival rate. Given the promising biological evaluation results, the authors highlighted that **36** behaves as a pro-drug with potential for the effective treatment of PR(+) breast cancer [97].

### 5.2. Targets Involved in Metabolic Pathways

An extensive number of inorganic and organometallic gold and ruthenium complexes have been conjugated to several different biomolecules for targeted anticancer purposes. These include systems targeting the amino acid transporters [102,106,107,108,109], peptide transporters [110,111], glucose transporters [69,112,113], and vitamin transporters [77,114,115,116,117,118]. As our review mainly focuses on the emerging receptors for the target delivery of ruthenium and gold complexes into cancer cells, for which the use of biomolecules as delivering carriers is well-known and is out of our scope, we will not discuss them in further detail. Nevertheless, for the sake of exemplification, we selected two ruthenium conjugates that showed promising in vivo results, one with a glucose targeting moiety and another with a biotin (vitamin B7) as vector. Moreover, in this subsection, we will also discuss a couple of gold conjugates, aiming for the glucose and the biotin transporters, which are structurally analogous to some compounds that we discussed before (**15** and **19**).

In contrast with normal cells whose metabolic activity relies primarily on mitochondrial oxidative phosphorylation to generate ATP for energy, the cancer cells overwhelmingly produce energy through the much less efficient process glycolysis. This is known as the Warburg effect, and to fulfil the high demand on glucose, cancer cells overexpress glucose transporters (GLUT) compared to the tissues that they are derived from [13]. Thus, the use of glucose and its derivatives for the targeted therapy, drug delivery, and especially the precise imaging of cancer is a well established approach [61].

**Figure 13:**
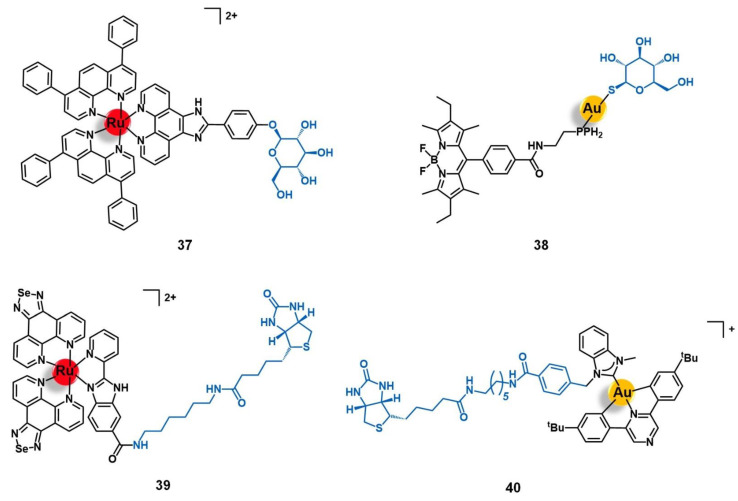
Ru(II), Au(I) and Au(III) conjugates that target the glucose receptors (37 and 38) or the biotin receptors (39 and 40).

Very recently, Chao and colleagues reported the conjugation of several luminescent Ru(II)-polypyridyl complexes to glucose aiming to develop a target two-photon PDT of cancer [113]. All resulting conjugates kept the original photophysical properties of the respective complexes after glucose tethering and showed a higher level of cell uptake in several different cancer cell lines. Among them, conjugate **37** (Figure 13) emerged as the most promising drug candidate. This conjugate was obtained by the conjugation of complex [Ru(dip)_2_(PIP)]^2+^ (dip = 4,7-diphenyl-1,10-phenanthroline; PIP = 2-(phenyl)imidazo[4,5-*f*]1,10-phenanthroline) to D-glucose at carbon C1. This position was selected as C1-substituted positional isomers typically have higher tolerance to bulky conjugates for keeping the affinity towards GLUT and to mimic the naturally occurring events during GLUT-mediated cell uptake. Conjugate **37** showed increasing cellular uptake by cancer cells relative to non-cancer cell lines under glucose starvation to mimic the tumor microenvironment, at the order HeLA cervical cancer > HepG2 liver cancer > A549 and A549 lung cancer > L02 non-tumoral hepatocytes. Indeed, a five-fold increase in the uptake of **37** was observed for the HeLa cancer cells compared to the L02 healthy cells. By contrast, the precursor complex [Ru(dip)_2_(PIP)]^2+^ barely showed any difference between the cancer cell lines with an overall lower level of internalization. Interestingly, while in the case of [Ru(dip)_2_(PIP)]^2+^, internalization in HeLa cancer cells was accomplished by passive diffusion, the uptake of **37** was primarily mediated by the glucose-transporter, with about 20% of it via GLUT and 80% mediated by the sodium-dependent glucose cotransporters (SGLT). Once inside the cells, the conjugate was mainly retained at the mitochondria (88%). Additionally, **37** also showed high PDT-induced cytotoxicity in the low micromolar range against the stated cancer cell lines, where it induced the generation of ROS and mitochondrial damage. This conjugate was more active against HeLa cells (IC_50_ = 2.1 µM), probably due to the higher cellular uptake, with a phototoxic index (light/dark) of 44 and selectivity index, compared to the non-tumoral L02 cells, of 10. As per the respective non-conjugated complexes, it was seven-fold less active in that cell line than the conjugate and showed a selectivity index lower than 2. In a HeLa xenograft model of BALB/c mice, PDT treatment with **37** resulted in a gradual shrinking of the tumors that ultimately disappeared, without signs of side effects for the mice or body weight loss. Based on these results, the authors stated that conjugate **37** show a very promising potential to be further developed as a cancer-specific two-photon PDT drug [113].

Analogously to the gold(I)-bombesin conjugate **15** (Figure 6), Bodio et al. also reported the conjugation of [Au(DPPEB-BODIPY)Cl] to a thiolate derivative of glucose for targeted anticancer applications [69]. The resulting conjugate (**38**, Figure 13) showed similar photophysical properties to those of the precursor complex (quantum yield of 86%), which suggests that, unlike bombesin conjugation (see Section 3.2), glucose conjugation does not affect the luminescent characteristics of the complex. Conjugate **38** showed higher cytotoxicity than **15** against prostate and breast cancer cell lines (IC_50_ PC-3 = 29.4 µM; IC_50_ MDA-MB-231 = 29.5 µM) but was not able to differentiate between those two. However, the glucose-conjugate was shown to be less toxic than the bombesin-conjugate against HMEC healthy mammary epithelial cells, with a more favorable MDA-BD-231/HMEC index (1.2) [69].

Tumor-targeting drug delivery systems based on the use of vitamins (e.g., biotin) as carriers of anticancer drugs have been intensively studied as well [119]. Analogously to the use of glucose-based vectors, this approach relies on the fact that cancer cells overexpress vitamin transporters, such as the sodium dependent multivitamin transporters (SMVT), compared to normal cells, as a mechanism of feedback to the high demand on nutrients needed for constant tumor growth and progression [120]. Envisioning the development of a new targeted anticancer theranostic agent for cervical carcinoma, Chen’s group reported the new Ru(II)-biotin conjugate **39** (Figure 13) that is responsive to the tumor microenvironment [118]. This compound was obtained by conjugation of the fluorescent and cytotoxic complex [Ru(phenSe)_2_(pbiz)]^2+^ (phenSe = [1,2,5] selenadiazolo[3,4-*f*] [1,10]phenanthroline) to a biotin through a hexamethylenediamine linker. Conjugate **39** showed favorable properties for bioimaging and a high stability in physiological conditions, but was pH sensitive. In slightly acidic aqueous conditions (pH ≈ 6.8), such as in the microenvironment around solid tumors, this conjugate hydrolyzes and releases the complex [Ru(phenSe)_2_(H_2_O)_2_]^2+^, which is responsible for the antiproliferative activity. Conjugate **39** is up to two-fold more active against several cancer cell lines (HeLa cervix, A549 lung, MCF-7 and MDA-MB-231 breast, and HepG2 liver) than against non-cancer cell lines (L02 liver, NCM460 colon). Interestingly, it was in the cervix cancer cells where **39** was simultaneously more cytotoxic (IC_50_ = 15.3 µM) and selective, being five-fold more active compared to the non-cancer L02 cells. The analogous active complex [Ru(phenSe)2(pbim)]^2+^ (pbim 2-(2-pyridyl)benzimidazole) showed a cytotoxicity similar to that of the conjugate but without selectivity towards any of the cancer cell lines tested. The uptake of conjugate **39** in HeLa cells is four-fold higher than in L02 cells and enters the cells via a biotin receptor-mediated endocytotic pathway. The non-conjugated complex was equally taken up by the two cell lines at a two-fold lower level than the respective conjugate. Once inside the cell, conjugate **39** induced mitochondrial dysfunctions with overproduction of ROS, resulting in apoptosis via induction of the endoplasmic reticulum stress signal pathway. After administration in HeLa-inoculated xenograft mice model, the conjugate accumulated preferentially in the tumor over other organs, with a three-fold higher accumulation compared to the non-conjugated complex, mainly retained in the liver and spleen, allowing tumor imaging and treatment. After a 30-day treatment, **39** significantly suppressed the progression of the tumor (64%) and reduced tumor-induced damage to normal organs of the mice, including liver, lung, and kidney. Therefore, the authors considered that conjugate **39** is a theranostic prodrug with desirable characteristics for its potential application in the targeted treatment and imaging of cancer [118].

Bertrand and co-workers also reported the conjugation complex [Au(bbfpz)(acbim)]^+^ to biotin (**40**, Figure 13), with the objective of developing a targeted therapy for cancers overexpressing the biotin receptors (BR) [77]. The uptake of conjugate **40** in BR(+) MCF-7 breast cancer cells was significantly higher than in BR(−) HCT-116 colorectal cancer cells. However, when the cytotoxicity was assessed against a panel of different BR(+) and BR(−) cancer cell lines, **40** did not show a significative difference among them (with IC_50_ values circa 10 µM). Indeed, in BR(+) A549 lung cancer cells, the conjugated had no activity at all (IC_50_ > 100 µM). Therefore, contrary to the analogous Au-estradiol conjugate **19** (Figure 8)**,** which is more active against cells that overexpress its receptor (see Section 3.4), the antiproliferative activity of **40** does not depend on the BR receptors. Moreover, conjugate **40** is even less active than the respective non-conjugated complex (up to 16-fold), which suggests that in this case, introduction of biotin moiety is not a good approach for vectorizing cytotoxic gold complexes to tumoral cells [77].

### 5.3. Cell Organelles and Targeted Gene Therapy

Apart from the molecular targets discussed above, cell organelles have also been widely studied and exploited as targets for the selective delivery of cytotoxic ruthenium and gold complexes to cancer cells. This approach relies on the slight differences between the organelles of the cancer cells and those of the non-tumoral cells, including, amongst others, overexpression of specific antigens, alteration of the physicochemical properties such as overall charge or electric potential, and mutations over specific genes [121,122]. One of the most exploited strategies to target organelles relies on the use of cell penetrating peptides (CPP; usually positively charged sequences such as polyarginines, oligolysines, or peptides derived from the trans-activating transcriptional protein of HIV-1 virus), mitochondrial penetrating peptides (MPP; e.g., FrFKFrFK), endoplasmic reticulum directing peptides (ERD; (e.g., penetratin peptide of sequence RQIKIWFQNRRMKWKK)), or nuclear localization sequences (NLS; e.g., PKKKRKV, KSKKQK, VQRKRQKLMP, and other peptides derived from the nuclear factor-kappa B protein)) that can redirect cargo to the cytoplasm, mitochondria, endoplasmic reticulum, or cell nucleus, respectively.

Several Ru(II)-polypyridyl complexes have been derivatized with CPPs, MPP, ERD, or NLS for targeted PDT of cancer [62,123,124,125,126,127,128,129]. Gold(I)-MPP conjugates with cytotoxic activity against multi-drug resistant breast cancer cells have been reported as well [130].

However, in a general way, the use of redirecting peptides does not confer a tumor-exclusive way to deliver the complexes, as the peptides are often used to simply increase the cellular uptake and/or specific subcellular distribution and accumulation, which could occur in non-tumoral cells as well. Given this reason and the existence of other reviews covering this particular subject [98,100], we will not discuss this further. Instead, we will focus on the use of vectors that selectively target the tumoral cell organelles (membrane, mitochondria, and genes) which, in the case of ruthenium and gold complexes, includes the use of target specific peptides, proteins, antibodies, and oligonucleotides.

**Figure 14:**
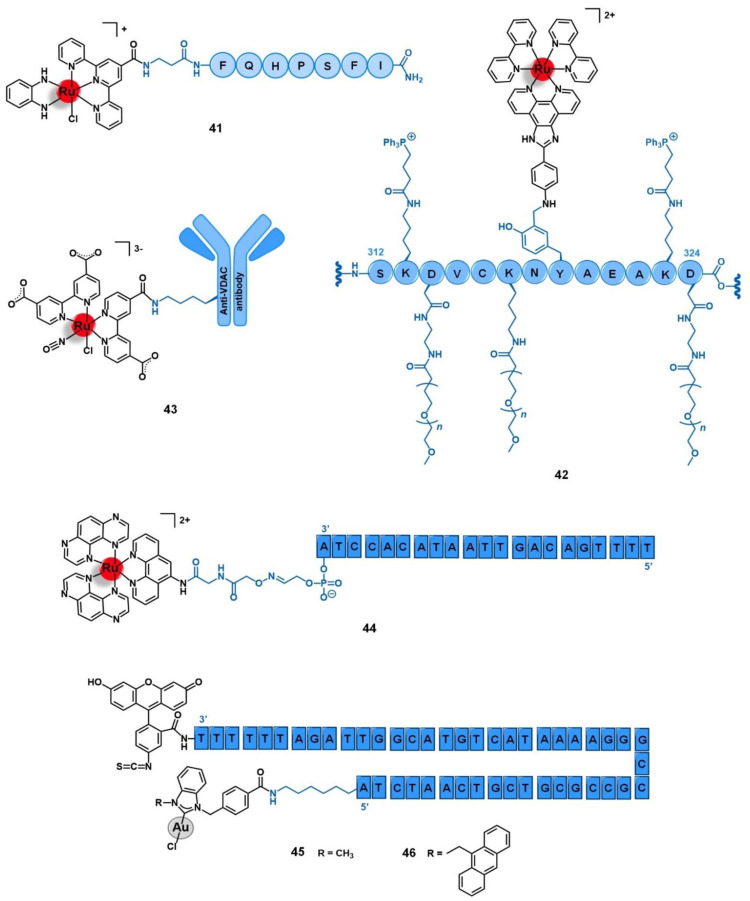
Ru(II) and Au(I) conjugates that target different organelles of cancer cells: **41** is specific for hepatoma cells membrane, **42** and **43** are specific of the mitochondria, and **44** to **46** include specific oligonucleotides for targeted delivery. Note that only a part of conjugate **42** is depicted (from amino acid 312 to 324 of the human serum albumin protein). VDAC = mitochondrial voltage-dependent anion-selective channels.

Zhang and co-workers have recently reported a ruthenium-peptide conjugate (**41**, Figure 14) for selective therapy of hepatocellular carcinoma [131]. This conjugate was obtained by conjugating [Ru(OPD)(terpyCOOH)Cl]^+^ (ODP = *ortho*-phenylenediamine) to peptide HCBP1 (FQHPSFI), using β-alanine as a spacer. The peptide HCBP1 is known to bind selectively to the surface of hepatoma cells, sparing normal hepatocytes as well as other cancer and healthy cells, and thus was selected as the targeting vector. Conjugate **41** showed a higher level of uptake by Hep-G2 hepatoma cells than the respective non-conjugated complex [Ru(OPD)(terpyCOOH)Cl]^+^ and an analogous conjugate with the inverse peptide sequence (IFSPHQF), highlighting the importance of HCBP1 as vector. Additionally, **41** also showed high and selective cytotoxicity towards hepatocellular carcinoma cells (IC_50_ Hep-G2 = 5.3 µM; IC_50_ Hepa-1G = 4.5 µM), being less active against HL-7702 non-tumoral liver cells and several other cancer cells lines (A2780 ovarian carcinoma, OE19 oesophageal carcinoma, HCT116 colorectal carcinoma, and PC-3 prostate carcinoma) with selectivity index values higher than 10 (IC_50_ > 40 µM). The non-conjugated complex and the analogous conjugate with the inverse-peptide sequence showed moderate-low cytotoxicity in the stated cell lines indiscriminately (with selectivity indexes < 1.5). Furthermore, in a 3D spheroid model of Hep-G2 cells, conjugate **41** also showed high cytotoxicity (IC_50_ = 9.6 µM), which together with the previous results prompted the authors to identify it as a promising candidate to be further developed as an anticancer agent for the precise therapy of liver cancer [131].

The vectorization of Ru(II)-polypyridyl complexes towards the mitochondria of cancer cells has been successfully accomplished using protein or antibody vehicles.

Aiming to develop a targeted two-photon PDT for acute myeloid leukemia, Weil et al. described the use of a human serum albumin (HSA)-based platform for selective delivery of the photosensitizer [Ru(bipy)_2_(NOP)]^2+^ (NOP = 4-(1H-imidazo [4,5-*f*] [1,10]phenanthroline-2-yl)-aniline) [132]. HSA was chosen not only because it accumulates efficiently at the cancer cells, where it enters via clathrin-mediated endocytosis, but also because it is an endogenous protein that is non-toxic and has well-established metabolic pathways. Conjugate **42** (Figure 14) was prepared by reaction of the ruthenium precursor complex with HSA previously modified both with a polyethylene oxide (PEO) polymer, for improving water solubility plus reducing non-specific interactions, and the mitochondria-targeting molecule triphenylphosphonium bromide (TPP), the conjugate being obtained at a ratio of 1 HSA:20 PEO:34 TPP:10[Ru(bipy)_2_(NOP)]^2+^. **42** kept both the biodegradable and the photoluminescent properties of the protein and the complex moieties, respectively. Upon irradiation, this conjugate was highly cytotoxic against several tumoral cell lines, namely HeLa cervical cancer (IC_50_ = 34.9 nM), MCF-7 breast cancer (IC_50_ = 114.2 nM), and A549 lung carcinoma (IC_50_ = 119.1 nM) while showing low antiproliferative activity in the dark. Compared to the non-conjugated complex [Ru(bipy)_2_(NOP)]^2+^, the conjugate showed an extraordinary 220-fold increase of activity against cervical cancer cells. Moreover, it was also able to efficiently block cell proliferation and the clonogenic potential of OCI-AML3 acute myeloid leukemic cells without affecting leukemic primary bone marrow cells. Given the good results, the authors identified this conjugate as a promising drug candidate for the selective treatment of leukemia [132].

Silva and co-workers developed a ruthenium-antibody conjugate (**43**, Figure 14) based on the nitric oxide (NO)-releasing complex [Ru(dcbipy)_2_(NO)Cl]^3−^ (dcbpy = 2,2′-bipyridine-4,4′-dicarboxylic acid) for the treatment of hepatocellular carcinoma [132]. NO is known both for its antiproliferative activity and for its pro-tumorigenic effects, depending on the concentration and the local of action. In cancer cells, NO can induce the generation of ROS and subsequent cell death, therefore external stimuli-dependent NO-donor complexes display promising anticancer potential by promoting a controlled release of the appropriate amount of NO into the target. Additionally, delivery systems that could vectorize this kind of complex directly towards the cancer cells could potentially avoid the off-site pro-tumorigenic activity against the healthy tissues. The authors showed that [Ru(dcbipy)_2_(NO)Cl]^3−^ is able to efficiently release NO under physiological conditions upon ligand exchange with water, induced by the mitochondria via an oxidation–reduction mechanism. Upon NO release, the mitochondrial respiratory chain is inhibited, which induces in turn the production of ROS and the formation of mitochondrial permeability transition pores, culminating in cell death. Conjugation of this complex with an anti-mitochondrial voltage-dependent anion-selective channels (VDAC) polyclonal antibody, through amide bond formation, resulted in conjugate **43** (Figure 14). The anti-mitochondrial VDAC antibody specifically targets the mitochondria outer-membrane surface marker VDAC that plays a key role in the generation of ROS and the mitochondria-mediated apoptosis, for which the authors expected a synergistic effect between the conjugate and the antibody in addition to the increase of selectivity. Conjugate **43** induced a decrease in the viability of the Hep-G2 hepatocellular carcinoma cell line up to 80%, while the unconjugated antibody and the free complex did not significantly change the cell survival rate. These results reinforce the potential of using antibodies as delivery carriers of cytotoxic ruthenium complexes directly to their biological targets [132].

The conjugation of Ru(II) and Au(I) complexes with oligonucleotides for targeted gene-therapy of cervical cancer and leukemia has been reported as well. Reschner and colleagues tethered the photoreactive complex [Ru(TAP)_2_(phenNH_2_)]^2+^ (TAP = 1,4,5,8-tetraaza phenanthrene; and phenNH_2_ = 1,10-phenanthrolin-5-amine) to the antisense oligonucleotide ASO-E6 (at 3′) through a diglycine-like linker for a targeted PDT-induced gene-silencing anticancer approach (**44**, Figure 14) [132]. The human papilloma viruses (HPV)-related cancers, such as the cervix carcinoma, are often associated with the expression of the HPV16 E6 oncogene, which blocks the p53 tumor suppressor pathway and induces tumorigenesis. Therefore, gene therapy approaches aiming to silence the E6 oncogene constitute attractive therapeutic routes to threaten this kind of cancer. In this approach, the delivering and active moiety of the conjugate corresponds to the oligonucleotide ASO-E6 (ATC CAC ATA ATT GAC AGT TTT) that targets the E6 gene at position 324, while the complex [Ru(TAP)_2_(phenNH_2_)]^2+^ confers the conjugate light-responsive for a controlled activation of the antisense gene therapy. Upon blue-light irradiation, **44** was able to regulate the E6 oncogene by irreversibly crosslinking the targeted sequence, resulting in a downregulation of E6 at the protein level and the reactivation of the p53. As a result, this conjugate induced the inhibition of cell proliferation of HPV16^+^ SiHa cervical cancer cells both in monolayer culture and in a three-dimensional model upon light irradiation (ca. 60% reduction), while showing no antiproliferative activity in the dark. Given the results, the authors considered that conjugate **44** holds potential to be further explored for the selective therapy of cervix cancer, based on a gene silencing approach activated under visible-light illumination [132].

Veige’s team reported the conjugation of two Au(I) *N*-heterocyclic carbene complexes to oligonucleotide sgc8c at 5′ (AGA TTG GCA TGT CAT AAA AGG GCC GCC GCG TCG TCA ATC TA) previously modified with a fluorescein isothiocyanate fluorophore (at 3′) through two spacers at each extremity (**45** and **46**, Figure 14) [132]. The oligonucleotide sgc8c (was chosen as vector because it is known to specifically target CCRF-CEM leukemia cells. Both conjugates showed higher cytotoxicity against this cell line than the respective precursor complexes, with **45** (IC_50_ = 0.5 µM) showing a 27-fold activity increase, and **46** (IC_50_ = 2.4 µM) a 13-fold. Conjugate **46** was selectively recognized and internalized into CCRF-CEM cells over another leukemia cell line (K562). Additionally, a library of analogue conjugates composed of random oligonucleotides did not show any antiproliferative activity in either of the two cancer cell lines, confirming the role of sgc8c as a vector for the selective delivery of cytotoxic gold complexes into CCRF-CEM leukemia cancer cells [132].

## 6. Conclusions

This review article summarizes the emerging molecular receptors and other cellular targets that have been exploited in the past years for the target-specific delivery of ruthenium and gold complexes into cancer cells, given the differences existing between tumoral and normal cells on the expression, structure, and/or function of those targets. Several drug delivery systems have been designed based on different types of targeting moieties, ranging from small molecules to antibodies, but the great majority relies on the use of peptides as vectors. The functionalization of the metal complexes with the targeting vectors has been accomplished either by direct coordination to the metal center or by conjugation to the complexes through a linker. A few examples of the main approaches developed were discussed.

Considering the novelty of this research topic, only a reduced number of studies has been reported, most of them in the early stage of preclinical evaluation with scarce information on in vivo behavior. In general, the incorporation of the delivery vector increases the selectivity of the complexes towards the cancer cells that overexpress the corresponding targets compared to normal cells. However, in many cases, the antiproliferative activity of the complexes was partially reduced or even completely lost when the conjugation of the targeting vector to the cytotoxic complex was irreversible. Indeed, the most promising results were attained for conjugates containing a cleavable linker, which allowed release of the active complex in the tumor microenvironment. Nonetheless, the specific delivery of ruthenium and gold complexes into cancer cells seems to be an attractive and promising approach to fulfil the current and future needs in cancer therapy.

Brought together, the data collected give an insight into some features that might be useful when designing novel ruthenium and gold anticancer agents for precision medicine.

## Figures and Tables

**Table 1:** Ru and Au metal complexes and their vectors developed for target membrane proteins: cell adhesion molecules, G-protein coupled receptors, and growth factor receptors.

Target		Vector	Metal Complex	Ref.
**Cell Adhesion Molecules**	Integrins	RGD	[Ru(h^6^-*p*-cym)(bpm)(pyac)]^2+^	[45]
		*cyclo*-RGDfK peptide	[Ru(terpy)(terpyCOOH)]^2+^ [Ru(terpyCOOH)_2_]^2+^ [Ru(phen)_2_(phenimi)]^2+^ [Ru(POP)_2_(pbiz)]^2+^ [Ru(terpy)(bipy)(D-biotin)]^2+^ [Au(ppy)(Lpa)]	[46,47,48,49,51,52]
		DfKRG peptide	[Au(ppy)(Lpa)]	[52]
	Cadherins	HAV peptide	^67^Ga-NODAGA- [(η^6^-Tyr-RuCp)]	[57]
**G Protein-Coupled Receptors**	Somatostatin receptors	AGCKNFFWKTFTSC peptide	[Ru(bipy)_3_]^2+^	[64]
		Cyclic-fCFwKTCT peptide	[Ru(η^6^-*p*-cym)(bpm)(pyac)]^2+^ [Ru(η^6^-*p*-cym)(dap)Cl]^+^ [Ru(η^6^-*p*-cym)(PPh_3_)(imbez)Cl]^+^	[45,66]
	Bombesin receptors	QWAVGHLM peptide	[Ru(dppz)_2_(CppH)]^2+^ [Au(DPPEB-BODIPY)Cl]	[69]
	Opioid receptors	leu-enkephalin peptide (YGGFL)	[Ru(η^6^-*p*-cym)(azapyr)Cl]	[72]
		met-enkephalin peptide (YGGFM)	[AuN_3_PPh_3_]+ [Ir(ppy)_2_(phenCOONa)]	[73]
	G protein-coupled estrogen receptors	17α-ethinylestradiol	[Au(bbfpz)(acbim)]^+^	[77]
		Tamoxifen	Ru(phen)_2_(phenimi)]^2+^	[78]
		Anastrozole	[Ru(η^6^-C_6_H_6_)(PPh_3_)(η^1^-ATZ)Cl]^+^	[79]
**Growth Factors Receptors**	Epidermal growth factor receptor	Tyrphostin (TYR) peptide	[Ru(η^6^-*p*-cym)(TYR)Cl_2_] [Ru(η^6^-toluene)(TYR)Cl_2_]	[84]
		4-anilinoquinazoline derivatives: AQZ, AcetAQZ	[Ru(η^6^-benzene)(enAQZ)Cl]^+^ [Ru(η^6^-*p*-cym)(enAQZ)Cl]^+^ [Ru(bipy)_2_(bipyCOOH)]^2+^	[85,86,87,88]
		erlotinib	[Au(erlotinib)(PPh_3_)]	[89]
	Human epidermal growth factor receptor 2	Trastuzumab antibody	[Au(PPh_3_)(DPTP)] [Au(PPh_3_)(MBANHS)]	[91]
		LTVSPWY peptide	[Au(ppy)(Lpa)]	[52]
	Fibroblast growth factor receptor	Peptides: GPPDWHWKAMTH SRRPASFRTARE VSPPLTLGQLLS	[Ru(CpCOOH)(bipy)(PPh_3_)]^2+^	96

## Data Availability

The data presented in this study are available on request from the corresponding authors.
